# Endosomal Trafficking Bypassed by the RAB5B‐CD109 Interplay Promotes Axonogenesis in *KRAS*‐Mutant Pancreatic Cancer

**DOI:** 10.1002/advs.202405092

**Published:** 2024-11-03

**Authors:** Dingwen Zhang, Yuming Luo, Yan Lin, Zhou Fang, Hanhao Zheng, Mingjie An, Qingyu Xie, Zhuo Wu, Chao Yu, Jiabin Yang, Min Yu, Changhao Chen, Rufu Chen

**Affiliations:** ^1^ School of Medicine South China University of Technology Guangzhou Guangdong 510006 P. R. China; ^2^ Department of Pancreatic Surgery Guangdong Provincial People's Hospital Guangdong Academy of Medical Sciences Guangzhou Guangdong 510080 P. R. China; ^3^ Department of Urology Sun Yat‐sen Memorial Hospital Sun Yat‐sen University Guangzhou Guangdong 510120 P. R. China; ^4^ Guangdong Provincial Key Laboratory of Malignant Tumor Epigenetics and Gene Regulation Sun Yat‐sen Memorial Hospital State Key Laboratory of Oncology in South China Guangzhou Guangdong 510120 P. R. China; ^5^ The Second School of Clinical Medicine Southern Medical University Guangzhou Guangdong 510080 P. R. China

**Keywords:** circular RNA, engineered extracellular vesicle, KRAS mutation, pancreatic cancer, perineural invasion

## Abstract

Perineural invasion (PNI) represents a unique biological feature associated with poor prognosis in pancreatic ductal adenocarcinoma (PDAC), especially in the presence of *KRAS* mutations. Extracellular vesicle (EV)‐packaged circular RNAs (circRNAs) function as essential mediators of tumor microenvironment communication, triggering PDAC cell invasion and distant metastasis. However, the regulatory mechanisms of EV‐packaged circRNAs in the PNI of *KRAS*‐mutant PDAC have not yet been elucidated. Herein, a *KRAS^G12D^* mutation‐responsive EV‐packaged circRNA, circPNIT, which positively correlated with PNI in PDAC patients is identified. Functionally, *KRAS^G12D^* PDAC‐derived EV‐packaged circPNIT promoted axonogenesis and PNI both in vitro and in vivo. Mechanistically, the circPNIT‐mediated Rab5B‐CD109 interplay bypassed traditional endosomal trafficking to anchor Rab5B to the lipid rafts of multivesicular bodies and packaged circPNIT into CD109^+^ EVs. Subsequently, CD109^+^ EVs delivered circPNIT to neurons by binding to TRPV1 and facilitating DSCAML1 transcription‐induced axonogenesis, which in turn enhanced the PNI by activating the GFRα1/RET pathway. Importantly, circPNIT‐loaded CD109^+^ EVs are established to dramatically promote PNI in a *KRAS^G12D/+^ Trp53^R172H/+^ Pdx‐1‐Cre* mouse model. Collectively, the findings highlight the mechanism underlying how EV‐packaged circRNAs mediate the PNI of *KRAS*‐mutant PDAC cells through the Rab5B endosomal bypass, identifying circPNIT as an effective target for the treatment of neuro‐metastatic PDAC.

## Graphical abstract

This study demonstrates a paracrine mechanism for EVs secretion mediated by Rab5B‐CD109 interplay in KRAS‐mutant pancreatic cancer, which triggers targeted delivery of circPNIT through CD109^+^EVs to neurons, thus promoting tumor‐associated axonogenesis and PNI, highlighting the CD109^+^ EV‐packaged circPNIT as an effective target for early intervention of PDAC patients with PNI.

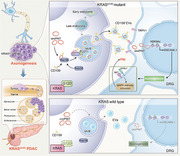

## 1 Introduction

Pancreatic ductal adenocarcinoma (PDAC) is a digestive malignancy with a high rate of *KRAS* oncogenic mutation,^[^ ^1^ ^]^ resulting in a 5‐year survival rate of less than 12%.^[^ ^2^ ^]^ Additionally, 90% of PDAC patients with *KRAS* mutations exhibit extensive perineural invasion (PNI) at an early stage, resulting in a nearly 23‐month reduction in their overall survival (OS).^[^ ^3^ ^]^ Thus, PDAC patients with *KRAS* mutations and PNI exhibit a highly aggressive phenotype. However, most of these patients often face increased risk owing to the lack of effective treatment options available in clinical decision‐making. Therefore, elucidating the relationship between *KRAS* mutations and PNI in PDAC and exploring the underlying regulatory mechanisms are of theoretical significance for improving the prognosis of PDAC.

PDAC with oncogenic *KRAS* mutations alters the tumor microenvironment to promote tumor metastasis by releasing extracellular vesicles (EVs),^[^ ^4^ ^]^ a diverse group of bilayer membrane structures. Inhibiting the formation, release, or uptake of EVs is anticipated to be a pivotal strategy for blocking the progression of *KRAS*‐mutant PDAC.^[^ ^5^ ^]^ Among these, EVs harboring oncogenic *KRAS^G12D^*‐specific short interfering RNAs (siRNAs) demonstrated sustained tumor‐suppressive effects in an orthotopic xenograft model of PDAC, addressing the challenge of targeting *KRAS* mutations.^[^ ^5a^ ^]^ Thus, EVs may be crucial regulatory mediators of metastasis, including PNI, in *KRAS*‐mutant PDAC. Consequently, investigating the regulatory mechanism of EVs is expected to lead to the identification of effective targets for the treatment of *KRAS* mutation‐induced PNI in PDAC patients.

Tumors deliver bioactive substances to specific target cells via EVs, thereby establishing a microenvironment conducive to the growth of disseminated cells.^[^ ^6^ ^]^ Therefore, blocking the secretion of tumor‐derived EVs is essential for inhibiting PDAC metastasis. The formation and secretion of EVs are complex biological processes that rely on endosomal maturation regulated by Rab GTPases and membrane fusion of multivesicular bodies (MVBs), which is mediated by the endosomal sorting complex required for transport (ESCRT).^[^ ^7^ ^]^ However, the mechanism by which *KRAS*‐mutant PDAC drives EV secretion has not yet been elucidated. Therefore, exploring EV secretion in *KRAS*‐mutant PDAC holds significant translational value, as it may reveal strategies to block PNI in PDAC patients.

In this study, we elucidated for the first time that *KRAS^G12D^*‐related PDAC cells exhibit severe PNI via the secretion of tumor‐derived CD109^+^ EVs, which deliver circPNIT to facilitate tumor‐associated axonogenesis and PNI. Mechanistically, circPNIT acts as a molecular scaffold, linking Rab5B to its novel effector, CD109, enabling specific anchoring on lipid rafts of the MVBs membrane. This interaction triggers lipid raft‐mediated EV secretion, revealing a bypass mechanism of endosomal trafficking mediated by the Rab5B‐CD109 interplay. Notably, CD109‐engineered EVs loaded with circPNIT effectively promoted neural invasion in a *KRAS^G12D/+^ Trp53^R172H/+^ Pdx‐1‐Cre* (KPC) mice model. Our study provides an applicable strategy for treating *KRAS^G12D^*‐mutated PDAC using PNI.

## 2 Results

### 2.1 KRAS^G12D^ Mutation is Associated with Axonogenesis and PNI in PDAC

**Figure 1:**
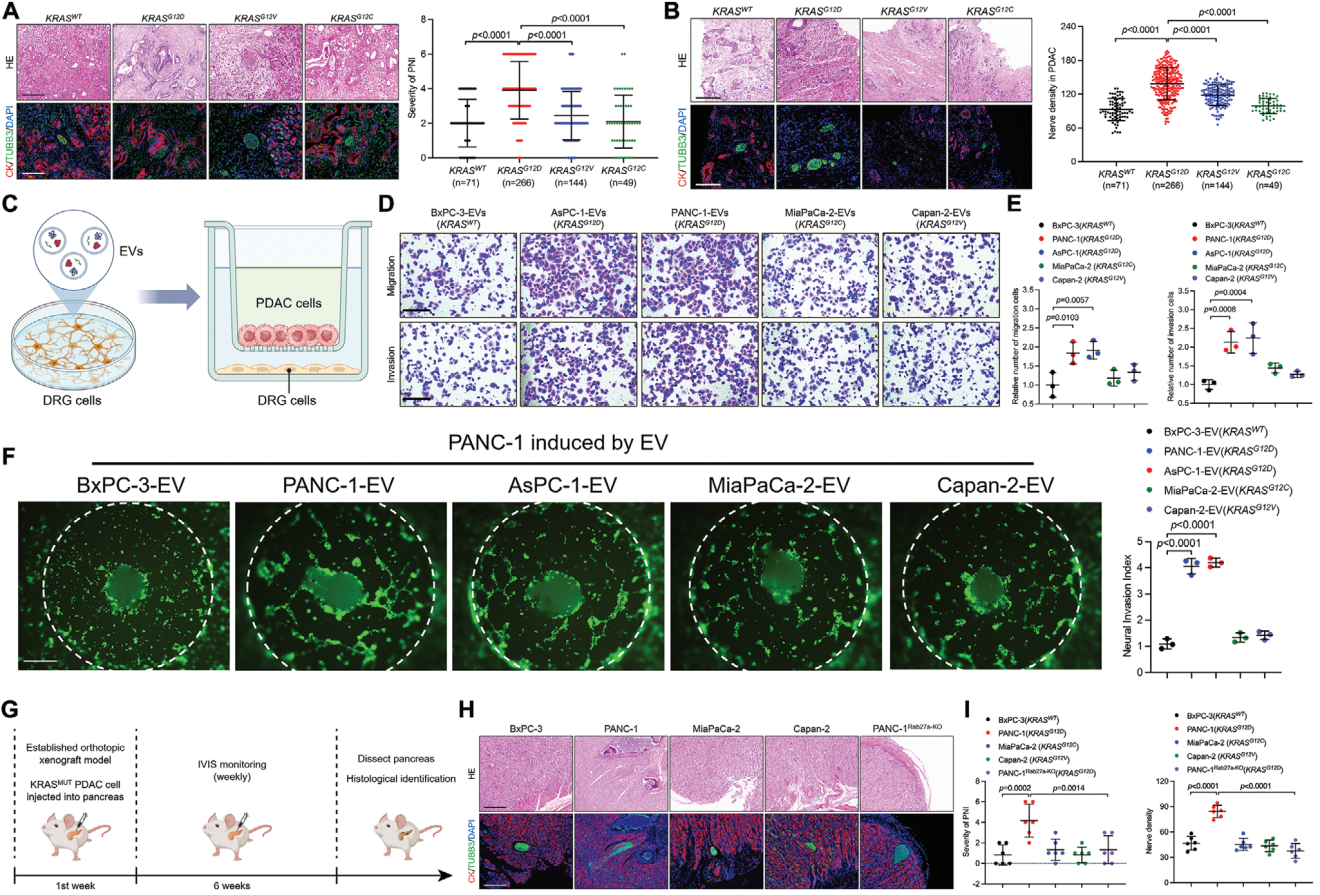
*KRAS^G12D^* mutation is associated with axonogenesis and PNI in PDAC. A‐B) Representative images and quantification of PNI severity (A) and nerve density (B) in PDAC specimen from patients with the *KRAS* mutation (*KRAS^WT^*, n = 71; *KRAS^G12D^*, n = 266; *KRAS^G12V^*, n = 144; *KRAS^G12C^*, n = 49). Scale bars: 50 µm. The nonparametric Mann‒Whitney *U* test was used. C) Schematic illustration of the PNI‐associated transwell assay. (D‐E) Representative images D) and quantification of neural invasion and migration E) of *KRAS* subtypes PDAC cells treated with EVs. Scale bar: 50 µm. One‐way ANOVA followed by Dunnett's test was used. F) Representative DRG matrix images and quantification of EV‐treated PDAC cells with the indicated KRAS subtypes. Scale bar: 50 µm. One‐way ANOVA followed by Dunnett's test was used. G) Schematic illustration of the establishment of the orthotopic xenograft model of the KRAS‐mutant PDAC cells. H‐I) Representative H&E staining and mIHC images (H), and quantification (I) of PNI severity and nerve density in KRAS‐mutant PDAC. Scale bars: 50 µm. One‐way ANOVA followed by Dunnett's test was used. The data are presented as the mean ± SD of three independent experiments. *P < 0.05, **P < 0.01.

To investigate the correlation between *KRAS* mutations and the PNI in PDAC, we evaluated *KRAS* subtypes in 530 PDAC patients from multiple centers using Sanger sequencing (Figure S1A, Supporting Information, *KRAS^WT^*: n = 71; *KRAS^G12D^*: n = 266; *KRAS^G12V^*: n = 144; *KRAS^G12C^*: n = 49). The PNI score based on multiplex immunohistochemistry (mIHC) assays showed that the severity of PNI was significantly higher in *KRAS^G12D^* PDAC patients than in those with other *KRAS* subtypes (*KRAS^WT^*, *KRAS^G12V^*, and *KRAS^G12C^*) (**Figures** **1A** and S1B, Supporting Information). Because tumor‐associated axonogenesis, which manifests as increased nerve density,^[^ ^8^ ^]^ represents the initial step that drives neuroplasticity and PNI, we conducted further analyses to determine whether *KRAS* is involved in axonogenesis in PDAC. Increased nerve density was observed in the *KRAS^G12D^* PDAC cohort compared to that in the other *KRAS*‐mutant subtypes (Figure 1B), indicating that the *KRAS^G12D^* mutation is associated with axonogenesis during PNI.

We have previously revealed the specific abundant production of EVs in response to the *KRAS^G12D^* mutation, which is involved in the early metastasis of PDAC.^[^ ^4b^ ^]^ Therefore, we hypothesized that *KRAS^G12D^*‐related PDAC cells trigger axonogenesis and PNI via EV transmission. To test this hypothesis, we isolated and characterized EVs from the culture media of PDAC cells with different *KRAS* subtypes (*KRAS^WT^*: BxPC‐3; *KRAS^G12D^*: PANC‐1, AsPC‐1; *KRAS^G12C^*: MiaPaCa‐2; *KRAS^G12V^*: Capan‐2). The isolated particle vesicles exhibited a typical double conical disc shape and ranged in size from 30 to 150 nm (Figure S2A–E, Supporting Information). Subsequently, to evaluate the effect of PDAC‐derived EVs on PNI, we found that compared with other *KRAS* subtypes, *KRAS^G12D^* PDAC cells exhibited enhanced neurotropism rather than invasiveness (Figures 1C–E and S2F–I, Supporting Information). Subsequently, we induced PANC‐1 through EV and EV‐free supernatant from *KRAS*‐mutant PDAC cells, which showed that PANC‐1 induced by EV‐free supernatant from *KRAS*‐mutant PDAC cells did not alter PNI function (Figure S2J,K, Supporting Information). Conversely, PANC‐1 cells induced by EVs from *KRAS^G12D^* PDAC exhibited enhanced neurotropism compared with EVs from other *KRAS* mutant PDACs (Figure 1F). Moreover, to confirm that EVs are indispensable for *KRAS^G12D^* PDAC‐induced PNI, we inhibited EV secretion from *KRAS^G12D^* cells by knocking out Rab27a. The results showed that the inhibition of EV secretion significantly decreased the neurotropism of PANC‐1 (Figure S2L–O, Supporting Information), indicating that EVs derived from *KRAS^G12D^* PDAC cells facilitate neurotropism in these cells.

To further explore the function of *KRAS* mutations in axonogenesis and PNI in PDAC in vivo, we established an orthotopic xenograft model via intrapancreatic injection of equivalent PDAC cells with different *KRAS* subtypes (Figure 1G; *KRAS^WT^*: BxPC‐3; *KRAS^G12D^*: PANC‐1; *KRAS^G12C^*: MiaPaCa‐2; *KRAS^G12V^*: Capan‐2). The PNI score indicated that *KRAS^G12D^*‐related PDAC was associated with increased nerve density and severity of PNI compared to other *KRAS* subtypes, whereas inhibition of EV secretion in Rab27a‐knock out (KO) mice suppressed the severity of neural infiltration in PANC‐1‐induced tumor tissues (Figures 1H–I and S2P, Supporting Information). Taken together, these results indicated that *KRAS^G12D^* PDAC‐induced EV significantly promoted axonogenesis and PNI.

### 2.2 Identification of circPNIT in KRAS^G12D^ PDAC‐Derived EVs and its Association with PNI

**Figure 2:**
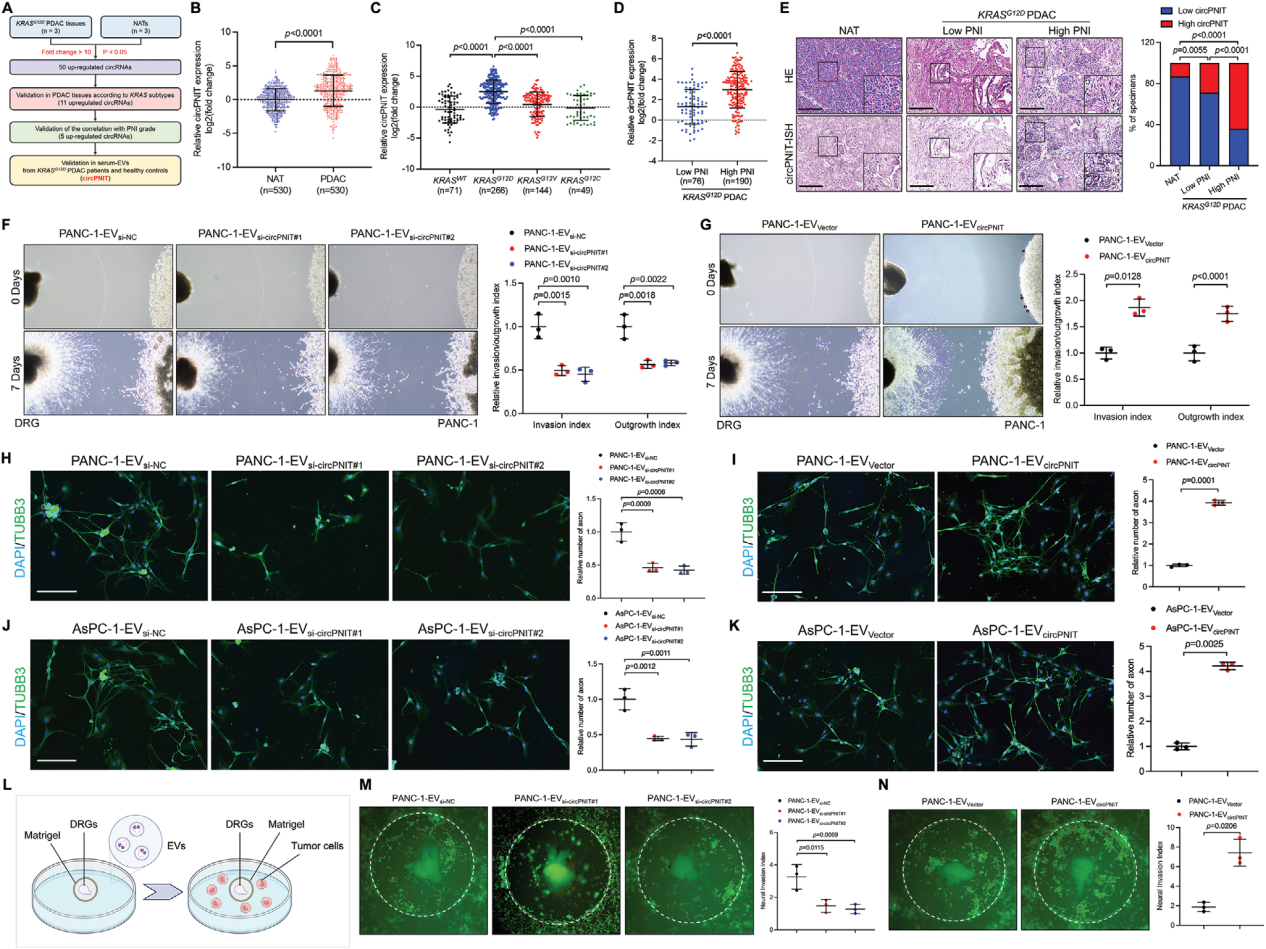
*KRAS^G12D^* PDAC‐derived EV‐packaged circPNIT enhances axonogenesis in vitro. A) Schematic representation of circPNIT screening in *KRAS^G12D^* PDAC tissues, high PNI PDAC tissues, and PDAC patient serum EVs. B‐D) The qRT‒PCR analysis of circPNIT expression in PDAC tissues (B), PDAC with *KRAS* mutation (*KRAS^WT^*: n = 71; *KRAS^G12D^*: n = 266; *KRAS^G12V^*: n = 144; *KRAS^G12C^*: n = 49) (C), and PDAC with different PNI severity (D). The nonparametric Mann‒Whitney *U* test was used. E) Representative H&E and ISH images and percentages of PNI in *KRAS^G12D^* PDAC patients according to the circPNIT expression level. Scale bars: 50 µm. Original magnification, × 4 (insets in E). The χ^2^ test was used to assess statistical significance. F‐G) Representative Matrigel/DRG images and quantification of neural invasion and outgrowth in PANC‐1 cells treated with the indicated EVs. Scale bar: 50 µm. One‐way ANOVA followed by Dunnett's test and a 2‐tailed Student's t test was used. H‐K) Representative images and quantification of nerve density in DRG cells treated with the indicated EVs. Scale bar: 50 µm. One‐way ANOVA followed by Dunnett's test and a 2‐tailed Student's t test was used. L) Schematic illustration of the EV‐induced DRG‐matrix assays. M‐N) Representative DRG‐matrix images and quantification of neural invasion by PANC‐1 cells treated with the indicated EVs. Scale bar: 50 µm. One‐way ANOVA followed by Dunnett's test and a 2‐tailed Student's t test was used. The data are presented as the mean ± SD of three independent experiments. *P < 0.05, **P < 0.01.

The aberrant synthesis of circular RNAs (circRNAs) has been proposed to be involved in *KRAS* mutation‐induced metastasis in PDAC.^[^ ^9^ ^]^ Thus, to identify the crucial circRNAs involved in *KRAS^G12D^* PDAC‐derived EV‐mediated PNI, we analyzed our previous next‐generation sequencing data (GSE234760) for three pairs of *KRAS^G12D^* PDAC and normal adjacent tissues (NATs) (**Figure** **2**A). A total of 50 circRNAs that were upregulated by more than 10‐fold were subjected to a larger cohort of 530‐case PDAC patients, and 11 circRNAs were upregulated in *KRAS^G12D^* PDAC compared to those in NATs or PDAC with other *KRAS* subtypes (Figures 2B,C and S3A–L, Supporting Information). Further evaluation of the correlation between these 11 circRNAs and PNI grade in *KRAS^G12D^* PDAC tissues revealed five circRNAs that were overexpressed in *KRAS^G12D^* PDAC tissues with high‐degree PNI (high PNI) compared to those with low‐degree PNI (low PNI) (Figures 2D and S3M,N, Supporting Information). Subsequently, we analyzed the expression of these circRNAs in the serum EVs of 266‐case *KRAS^G12D^* PDAC patients and paired healthy volunteers, confirming that hsa_circ_0007006 (a term for perineural invasion‐associated transcript, circPNIT) was significantly upregulated in EVs obtained from *KRAS^G12D^* PDAC patients (Figures S3O–Q and S4A, Supporting Information). Therefore, circPNIT was selected for further analysis.

Subsequently, we explored the molecular characteristics of circPNITs. CircPNIT is derived from the back‐splicing of exons 10 to 13 of the DYM gene, which has a length of 514 nucleotides (nt), as confirmed via Sanger sequencing (Figure S4B, Supporting Information). In addition, circPNIT was amplified using only spliced cDNA instead of the gDNA template (Figure S4C, Supporting Information). Furthermore, we amplified circPNIT from the reverse‐transcribed sequence using random primers rather than oligo‐dT primers, confirming that circPNIT has a circular structure without a poly A tail (Figure S4D, Supporting Information). Moreover, compared to DYM mRNA, circPNIT exhibited stronger resistance to RNase R, and the actinomycin D assay confirmed that circPNIT has a longer half‐life than DYM mRNA (Figure S4E–G, Supporting Information). These results demonstrated that circPNIT possesses a stable circular structure.

Moreover, analysis of the pathological characteristics of *KRAS^G12D^* PDAC patients revealed a positive correlation between circPNIT expression and PNI severity (Table S1, Supporting Information). circPNIT expression was significantly upregulated in *KRAS^G12D^* PDAC tissues with high PNI and slightly increased in those with low PNI compared to NATs (Figure 2E). Furthermore, correlation analysis revealed that high circPNIT expression in *KRAS^G12D^* PDAC tissues was significantly positively correlated with nerve density (Figure S4H, Supporting Information). Additionally, Kaplan–Meier analysis of *KRAS^G12D^* PDAC patients revealed that high circPNIT expression was associated with shorter OS and disease‐free survival (DFS) (Figure S4I,J, Supporting Information). These results indicated that circPNIT was overexpressed in *KRAS^G12D^* PDAC‐derived EVs and positively associated with PNI in *KRAS^G12D^* PDAC patients.

### 2.3 KRAS^G12D^ PDAC‐Derived EV‐Packaged circPNIT Enhances Axonogenesis and PNI In Vitro

After demonstrating the enrichment of circPNIT in EVs and its clinical correlation with axonogenesis and PNI in *KRAS^G12D^*‐related PDAC, we further investigated the functional role of EV‐packaged circPNIT in *KRAS^G12D^*‐related PDAC‐induced axonogenesis and PNI. First, we found that applying siRNAs targeting circPNIT or circPNIT overexpression plasmids to *KRAS^G12D^* PDAC cells efficiently reduced or increased circPNIT expression in EVs, respectively (Figure S5A,B, Supporting Information). To verify the tumor‐nerve interaction, we constructed a Matrigel/dorsal root ganglia (DRG) model. The results showed that the migration and extension of neural axons were inhibited after culturing with EVs from circPNIT‐knockdown *KRAS^G12D^* PDAC cells, and these effects were accompanied by decreased neurotropism in PDAC cells (Figures 2F and S5C, Supporting Information). Conversely, incubation with EVs from circPNIT‐overexpressing *KRAS^G12D^* PDAC cells enhanced neural axon migration and extension in combination with increased neurotropism in PDAC cells compared to incubation with control EVs (Figures 2G and S5D, Supporting Information), indicating a positive relationship between axonal changes and neural invasion conferred by *KRAS^G12D^* PDAC‐derived EV‐packaged circPNIT. To determine the effects of EV‐packaged circPNIT on neural axons, we cultured DRG cells with *KRAS^G12D^* PDAC‐derived EVs and analyzed neuronal axonogenesis. The nerve density of DRG cells decreased after treatment with EVs from circPNIT‐knockdown *KRAS^G12D^* PDAC cells, whereas culturing with EVs from circPNIT‐overexpressing *KRAS^G12D^* PDAC cells increased the nerve density of DRG cells, indicating the positive effect of *KRAS^G12D^* PDAC‐derived EV‐packaged circPNIT on axonogenesis (Figure 2H–K). Further validation was performed using DRG matrix assays to explore whether axonogenesis mediated by EV‐packaged circPNIT was involved in the PNI of *KRAS^G12D^*‐related PDAC (Figure 2L). The results showed that the invasion of PDAC cells into the DRG was inhibited after treatment with EVs of circPNIT‐knockdown *KRAS^G12D^* PDAC cells compared to treatment with control EVs, whereas treatment with EVs from circPNIT‐overexpressing *KRAS^G12D^* PDAC cells significantly enhanced the neurotropism of *KRAS^G12D^* PDAC cells (Figures 2M,N and S5E,F, Supporting Information). Taken together, these results indicated that EV‐packaged circPNIT triggered PNI in *KRAS^G12D^* PDAC cells by promoting axonogenesis in vitro.

### 2.4 EV‐Packaged circPNIT Promotes Neural Metastasis of KRAS^G12D^‐Related PDAC In Vivo

**Figure 3:**
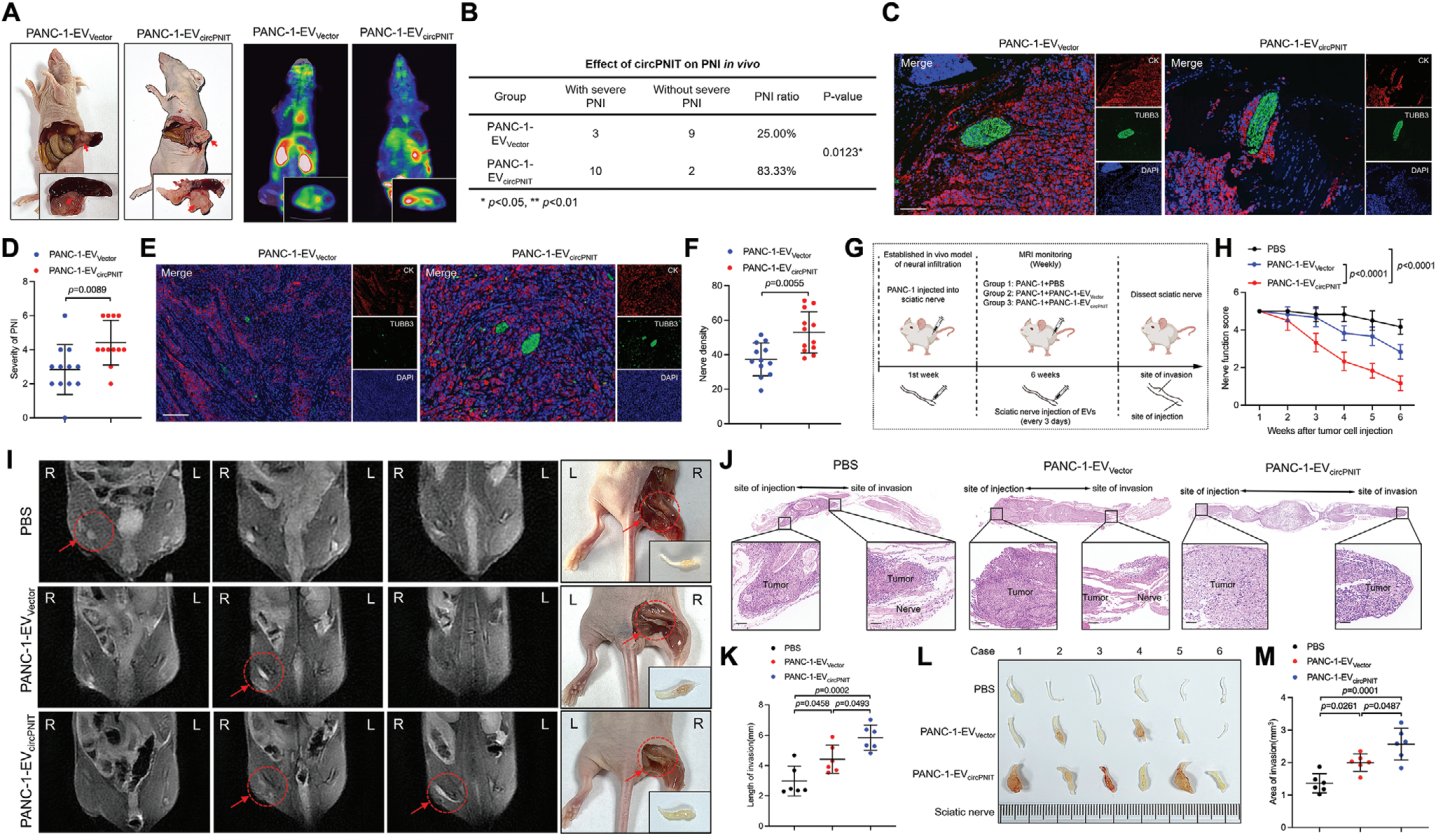
circPNIT packaged in EVs promoted the neural metastasis of *KRAS^G12D^* PDAC cells in vivo. A) Representative images of the EV‐induced orthotopic xenograft model and PET‐CT image. B) The PNI ratio of the primary tumor tissues treated with indicated EVs (n = 12 per group). The statistical differences between the groups were calculated using the χ^2^ test. C‐D) Representative mIHC images (C) and quantification of neural invasion (D) in PANC‐1 cells treated with the indicated EVs. Scale bar: 50 µm. A 2‐tailed Student's *t* test was used. E‐F) Representative mIHC images (E) and quantification of nerve density (F) in PANC‐1 cells treated with the indicated EVs. Scale bar: 50 µm. A 2‐tailed Student's *t* test was used. G) Schematic illustration of the establishment of the EV‐induced in vivo model of neural infiltration. H) The nerve function scores of nude mice inoculated with PANC‐1 cells and treated with PBS or the indicated EVs (n = 6 per group). One‐way ANOVA followed by Dunnett's test was used. I) Representative images of neural invasion monitored by MRI; the circles indicate tumors. J‐K) Representative H&E images (J) and quantification (K) of neural invasion were determined by measuring the length and area between the injection and invasion sites. Scale bar: 50 µm. One‐way ANOVA followed by Dunnett's test was used. L‐M) Representative images (L) and quantification (M) of sciatic nerve volume. Scale bar: 1 cm. One‐way ANOVA followed by Dunnett's test was used. The data are presented as the mean ± SD of three independent experiments. *P < 0.05, **P < 0.01.

To explore whether EV‐packaged circPNIT is involved in the PNI of *KRAS^G12D^* PDAC cells in vivo, we established an orthotopic xenograft model and treated mice with control or circPNIT‐overexpressing EVs (**Figures** **3**A and S5G,H, Supporting Information; PANC‐1‐EV_Vector_ or PANC‐1‐EV_circPNIT_). PNI score analysis showed that the severity of neural invasion within the primary tumor was significantly higher in the PANC‐1‐EV_circPNIT_ group than in the PANC‐1‐EV_Vector_ group (Figure 3B–D). Additionally, the density of nerves increased dramatically in PANC‐1‐EV_circPNIT_‐induced primary tumors (Figure 3E,F), suggesting that EV‐packaged circPNIT is involved in axonogenesis and PNI in *KRAS^G12D^*‐related PDAC cells. To further explore the effect of EV‐packaged circPNIT‐induced neurons on the neural metastasis of *KRAS^G12D^* PDAC cells, we constructed an in vivo model of neural infiltration to simulate the process of PDAC cell invasion along the nerves, which facilitated quantification of the distance PDAC cells invaded into the nerves (Figure 3G). An equal amount of PANC‐1 was injected into the sciatic nerves of the nude mice. After 1 week, the mice were randomly divided into three groups (six mice per group), and EV was injected into the sciatic nerve every 3 days (PBS, PANC‐1‐EV_Vector_, or PANC‐1‐EV_circPNIT_). The nerve function score confirmed that nude mice in the PANC‐1‐EV_circPNIT_ group showed significant dysfunction in the affected hind limb compared to the PBS and PANC‐1‐EV_Vector_ groups (Figure 3H). Moreover, magnetic resonance imaging (MRI) and HE analyses confirmed that PANC‐1‐EV_circPNIT_ enhanced the capacity of *KRAS^G12D^* PDAC to metastasize from the injection site along the sciatic nerve compared to the PBS and PANC‐1‐EV_Vector_ groups (Figure 3I–K). Furthermore, the volume of the invaded sciatic nerve was significantly higher in the PANC‐1‐EV_circPNIT_ group than that in the PBS and PANC‐1‐EV_Vector_ groups (Figure 3L,M). Taken together, these results suggested that EV‐packaged circPNIT facilitates axonogenesis and PNI in *KRAS^G12D^*‐mutant PDAC in vivo.

### 2.5 circPNIT Binds to Rab5B and CD109 to form a Ternary Complex

To explore the mechanism underlying the package of circPNIT into *KRAS^G12D^* PDAC cell‐derived EVs to induce PNI, we first analyzed the subcellular location of circPNIT in *KRAS^G12D^* PDAC cells by performing fluorescence in situ hybridization assays, which showed that circPNIT was mainly localized in the cytoplasm of *KRAS^G12D^* PDAC cells (Figure S6A,B, Supporting Information). Cytoplasmic circRNAs predominantly serve as miRNA sponges to suppress the AGO2‐dependent miRNA‐mediated degradation of downstream mRNAs and exhibit biological functions.^[^ ^10^ ^]^ Nevertheless, we observed that altering the expression of circPNIT in AGO2‐silenced *KRAS^G12D^* PDAC cells affected the expression of circPNIT in the corresponding EVs, which was consistent with the results in *KRAS^G12D^* PDAC cells without the AGO2‐KO (Figure S6C, Supporting Information). Moreover, circPNIT overexpression in EVs derived from AGO2‐silenced *KRAS^G12D^* PDAC cells facilitated axonogenesis (Figure S6D,E, Supporting Information), suggesting that circPNIT acts as an miRNA sponge to regulate its loading into *KRAS^G12D^* PDAC‐derived EVs and subsequently induces axonogenesis.

**Figure 4:**
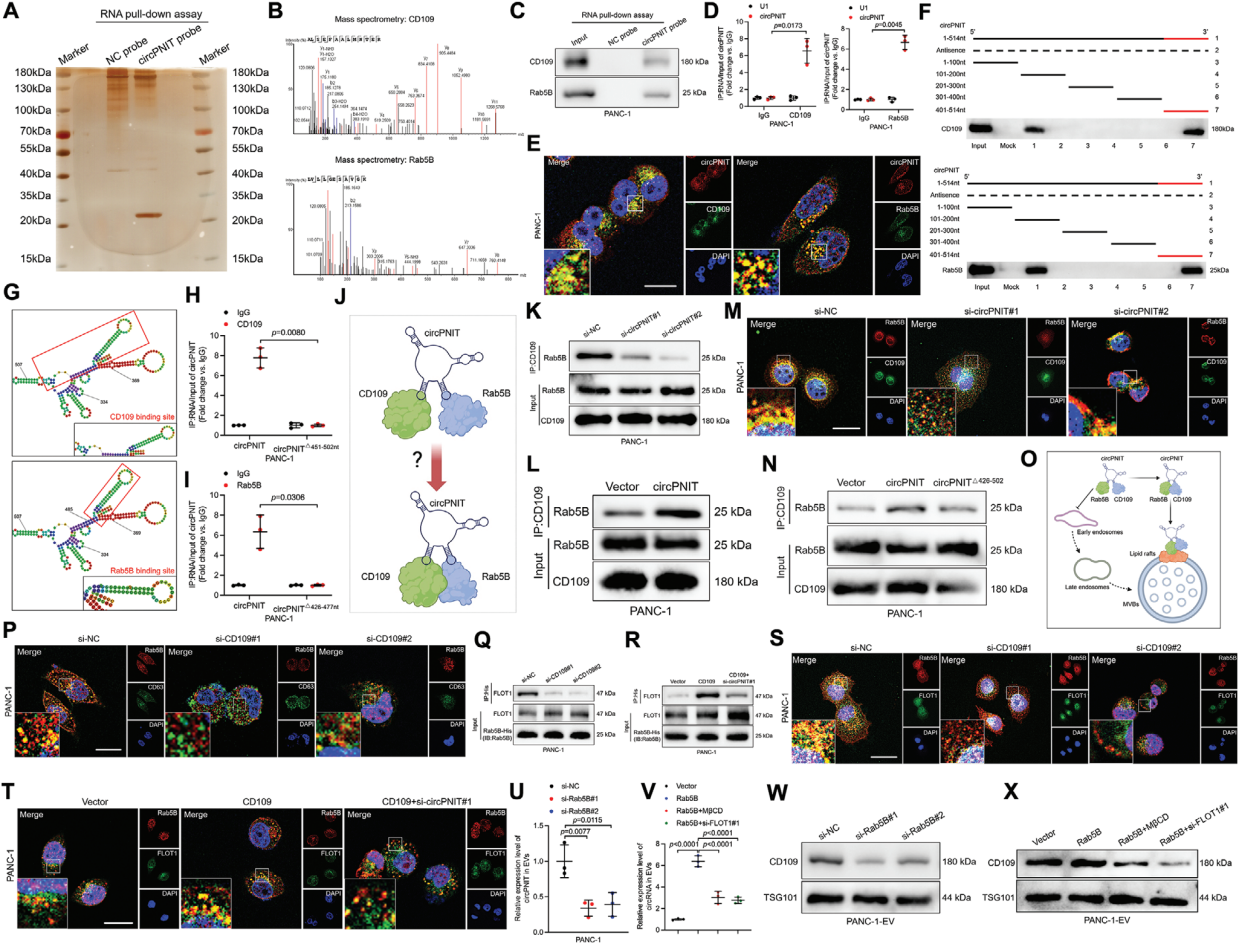
circPNIT binds to Rab5B and CD109 to form a ternary complex. A,B) Silver staining and mass spectrometry of the proteins from the RNA pull‐down assay. C) Western blot analysis confirmed that circPNIT was associated with CD109, and Rab5B. D) RIP assays confirmed that circPNIT interacted with CD109 and Rab5B. Negative control: IgG; nonspecific control: U1. E) Immunofluorescence was performed to assess the colocalization of circPNIT, CD109, and Rab5B in PANC‐1 cells. Scale bars: 5 µm. F) Sequential deletion assays confirmed that 401–514 nt of circPNIT is essential for binding Rab5B to CD109. G) Prediction of the stem‐loop structures of the CD109/Rab5B binding sites in circPNIT on the basis of CatRapid, a website for indicating the binding sites between non‐coding RNA and targeting proteins. H‐I) RIP assay showed that circPNIT enrichment of CD109 and Rab5B was effectively inhibited by mutations of 451–502 nt and 426–477 nt in circPNIT. J) Schematic illustration of the mechanism by which circPNIT promotes the binding of CD109 to Rab5B. K‐M) Co‐IP K and L) and IF M) assays analyzing the interaction between circPNIT‐mediated CD109 and Rab5B in PANC‐1 cells. N) Co‐IP assays analyzing the interaction between CD109 and Rab5B after the circPNIT^426‐502nt^ mutation in PANC‐1 cells. O) Schematic representation of the circPNIT/CD109/Rab5B complex localization in MVBs. P) Representative IF images showing CD63 and Rab5B colocalization mediated by CD109 in PANC‐1 cells. Scale bar: 5 µm. Q‐R) Co‐IP revealing the interaction of His‐labeled Rab5B with FLOT1 in PANC‐1 cells. S‐T) IF assays were performed to evaluate the localization between Rab5B and FLOT1, which was regulated by CD109 and circPNIT. U) Assessment of circPNIT expression in PANC‐1 cell‐secreted EVs after knockdown of Rab5B. V) Evaluation of circPNIT expression in PANC‐1 cell‐secreted EVs after treated by MβCD and si‐FLOT1. W) Analysis of CD109 expression in PANC‐1 cell‐secreted EVs after Rab5B was knocked down. X) Evaluation of CD109 expression in EVs secreted by Rab5B‐overexpressing PANC‐1 cells after lipid raft inhibition. Statistical significance was assessed using a 2‐tailed Student's t test (Figures D, H, and I) and one‐way ANOVA followed by Dunnett's test (Figures U and V). The data are presented as the mean ± SD of three independent experiments. *P < 0.05, **P < 0.01.

As the interaction of circRNAs with proteins is another important regulatory mechanism, we further investigated the binding partners of circPNIT in *KRAS^G12D^* PDAC. RNA pull‐down assays with biotinylated circPNIT revealed two prominent bands at 130–180 kDa and 20–35 kDa (**Figure** **4**A), which were confirmed to be CD109 and Rab5B, respectively, through mass spectrometry and western blot analysis (Figures 4B,C and S6F, Supporting Information). Fluorescence staining revealed the colocalization of circPNIT, CD109, and Rab5B in the cytoplasm of *KRAS^G12D^* PDAC cells (Figure 4E). Consistently, RNA immunoprecipitation (RIP) assays showed that circPNIT was enriched with CD109 and Rab5B (Figures 4D and S6G, Supporting Information), confirming that circPNIT interacts with CD109 and Rab5B, respectively. To further identify the binding sites of CD109 and Rab5B on circPNIT, sequential deletion assays were performed with truncated circPNIT sequences, which revealed that both CD109 and Rab5B were enriched in the 401–514 nt region of circPNIT (Figure 4F). Furthermore, sequence analysis using CatRapid predicted the potential binding site of CD109 in the 451–502 nt region of circPNIT and the potential Rab5B binding site in the 426–477 nt region of circPNIT (Figure 4G). Mutation of these two sites in circPNIT significantly reduced circPNIT enrichment by CD109 and Rab5B (Figures 4H,I and S6H, Supporting Information), indicating that these specific sequences are essential for circPNIT interaction with CD109 and Rab5B.

Considering the overlap in the binding sites of CD109 and Rab5B on circPNIT, we explored whether circPNIT was involved in the interaction between CD109 and Rab5B (Figure 4J). Co‐immunoprecipitation (co‐IP) assays showed that circPNIT knockdown significantly inhibited the interaction between CD109 and Rab5B, whereas circPNIT overexpression promoted CD109 binding to Rab5B in *KRAS^G12D^* PDAC cells (Figures 4K,L and S6I,J and S6L–N, Supporting Information). Moreover, fluorescence staining revealed the colocalization of CD109 and Rab5B in the cytoplasm of *KRAS^G12D^* PDAC cells, which was abolished by circPNIT knockdown (Figure 4M). Importantly, mutation of the 426–502 nt region in circPNIT impaired the interaction between CD109 and Rab5B (Figures 4N and S6K, Supporting Information), indicating that binding with circPNIT is crucial for the CD109‐Rab5B interaction. Taken together, these results indicated that circPNIT formed a stable ternary complex by linking Rab5B and CD109 in *KRAS^G12D^* PDAC cells.

### 2.6 circPNIT/CD109/Rab5B Mediates the Packaging of circPNIT into EVs

Given that Rab proteins regulate vesicle targeting, docking, and fusion by binding to specific effectors,^[^ ^11^ ^]^ we explored whether CD109 functions as an effector of Rab5B via circPNIT (Figure 4O). Immunofluorescence assays showed that blocking CD109 decreased the localization of Rab5B in CD63‐labeled MVBs (Figure 4P) and promoted the localization of Rab5B to early endosomes (EEA1‐positive), late endosomes (Rab7‐positive), and lysosomes (LAMP2‐positive), whereas circPNIT overexpression contributed to Rab5B localization in MVBs (Figure S7A,B, Supporting Information). Moreover, knockdown of CD109 impaired the localization of Rab5B and CD63 induced by circPNIT overexpression (Figure S7C, Supporting Information), indicating that circPNIT‐linked CD109 promoted the localization of Rab5B to MVBs. To clarify the binding of Rab5B to CD109, we constructed dominant active (Rab5B^Q79L^) and negative (Rab5B^D136H^) variants of Rab5B and verified the combination of Rab5B and CD109 in the active and negative states.^[^ ^12^ ^]^ The results showed a higher enrichment of CD109 in Rab5B^Q79L^ than in Rab5B^D136H^ (Figure S6O, Supporting Information). Conversely, si‐circPNIT effectively reduced CD109 enrichment induced by Rab5B^Q79L^ (Figure S6P, Supporting Information), indicating that Rab5B binding to CD109 occurs in a RabGTP‐activated state and is regulated by circPNIT. Considering that CD109 is a glycosylphosphatidylinositol‐linked glycoprotein that is mainly enriched in lipid rafts,^[^ ^13^ ^]^ we investigated whether lipid rafts are required for CD109‐mediated Rab5B localization in MVBs. Disrupting lipid rafts structure using methyl‐β‐cyclodextrin^[^ ^14^ ^]^ reversed the colocalization of Rab5B and CD63 induced by CD109 overexpression (Figure S7D, Supporting Information). Flotillin 1 (FLOT1), the core constitutive protein in lipid rafts,^[^ ^15^ ^]^ was further examined to determine CD109‐mediated Rab5B localization in MVB lipid rafts. Co‐IP assays showed that knockdown of CD109 significantly inhibited the ability of Rab5B to recognize FLOT1 (Figures 4Q and S7E, Supporting Information), whereas overexpression of CD109 promoted the interaction between Rab5B and FLOT1, which was reversed by circPNIT knockdown (Figures 4R and S7F, Supporting Information). Furthermore, disruption of the colocalization between Rab5B and FLOT1‐tagged lipid rafts was observed in *KRAS^G12D^* PDAC cells treated with CD109 or circPNIT knockdown (Figure 4S,T). These findings demonstrated that circPNIT‐linked CD109 mediated the translocation of Rab5B to MVBs through interactions with lipid rafts.

Next, we investigated whether the localization of Rab5B to MVBs contributes to the packaging of circPNIT into EVs. The results showed that Rab5B knockdown inhibited circPNIT expression in EVs, whereas Rab5B overexpression promoted circPNIT enrichment in EVs (Figures 4U and S7G, Supporting Information). Inhibiting lipid rafts by knocking down FLOT1 or MβCD significantly decreased the expression of circPNIT in EVs (Figure 4V), confirming that Rab5B mediated the loading of circPNIT into EVs in a lipid raft‐dependent manner. Considering that formation of the circPNIT/CD109/Rab5B complex triggers trafficking to MVBs and subsequent EV production, we further evaluated whether CD109 is also located in EVs. The inhibition of Rab5B significantly reduced the expression of CD109 in EVs (Figure 4W), whereas Rab5B overexpression promoted CD109 in EVs (Figure S7H, Supporting Information). In addition, blockade of lipid rafts by FLOT1 knockdown and MβCD reduced CD109 secretion in EVs (Figure 4X). Collectively, these results indicated that the circPNIT/CD109/Rab5B complex mediated the packaging of circPNIT and CD109 into EVs.

### 2.7 EV‐Packaged circPNIT Specifically Targets Neurons via CD109

**Figure 5:**
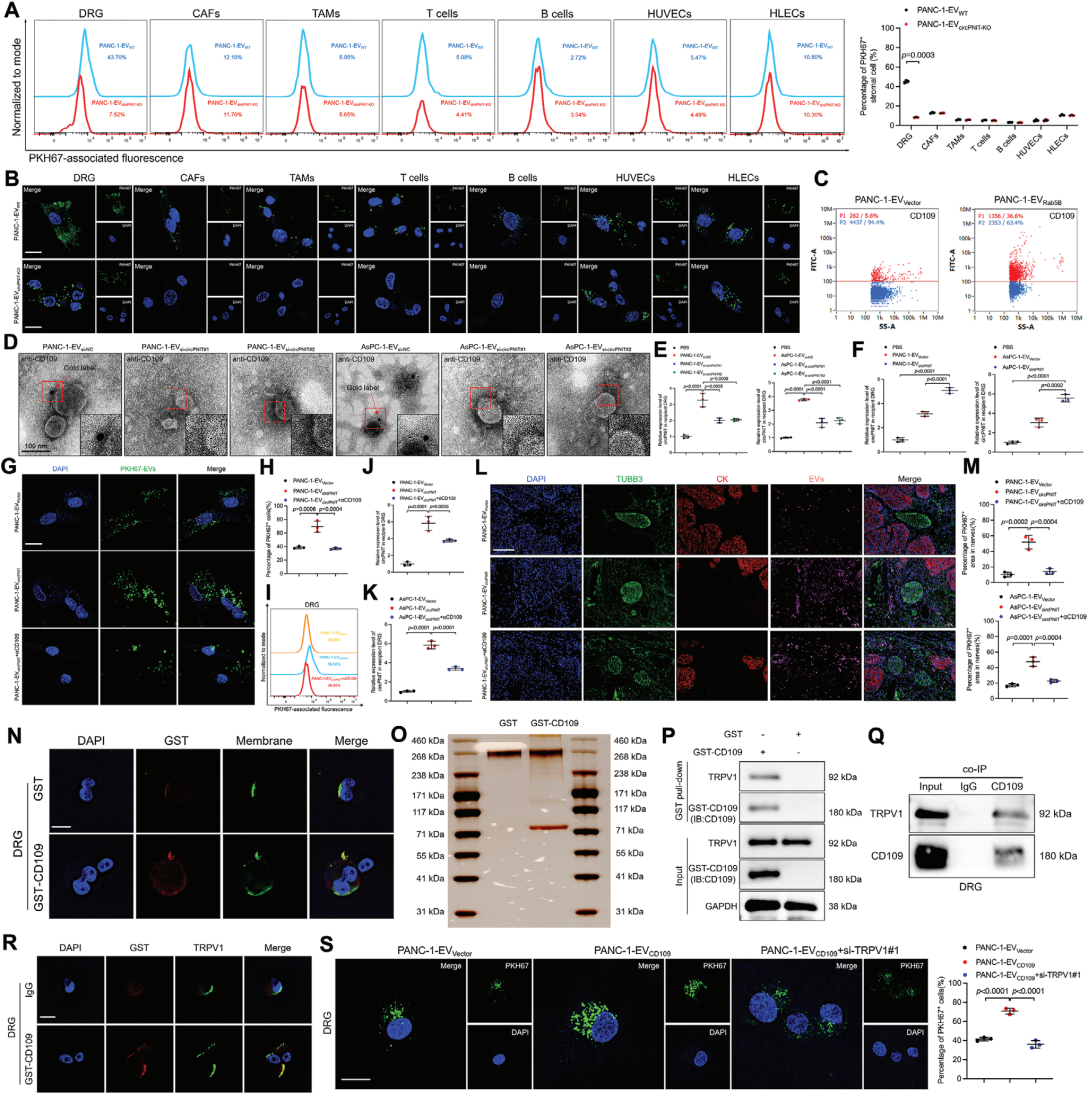
EV‐packaged circPNIT specifically targets neurons via the CD109‐TRPV1 interplay. A) Flow cytometric analysis and quantification of EVs taken up by stromal cells in the tumor microenvironment of PKH67‐labeled PANC‐1‐EV_WT_ or PANC‐1‐EV_circPNIT‐KO_ cells. B) Representative IF images of stromal cells treated with PKH67‐labeled PANC‐1‐EV_WT_ or PANC‐1‐EV_circPNIT‐KO_ cells. Scale bar: 5 µm. C) Nanoflow cytometric analysis confirmed the expression of CD109 in EVs derived from PANC‐1‐EV_Vector_ or PANC‐1‐EV_Rab5B_ cells. D) Representative electron microscopy image of EVs secreted by PANC‐1 and AsPC‐1 cells as indicated. E‐F) qRT‒PCR analysis of circPNIT expression in DRG treated with the indicated EVs. G‐H) Representative IF images (G) and quantification (H) of DRG cells treated with PKH67‐labeled PANC‐1‐EV_Vector_, PANC‐1‐EV_circPNIT_ or PANC‐1‐EV_circPNIT_+αCD109. Scale bar: 5 µm. I) Flow cytometric analysis of DRG cells treated with PKH67‐labeled PANC‐1‐EV_Vector_, PANC‐1‐EV_circPNIT_, or PANC‐1‐EV_circPNIT_+αCD109 cells. J‐K) qRT‒PCR analysis of circPNIT expression in DRG cells treated with the indicated EVs. L‐M) Representative mIHC images (L) and quantification (M) of EVs in the perineural region from primary tumor tissues treated with the indicated EVs. Scale bar: 50 µm. N) Representative PLA images showing the localization of GST‐CD109 on the surface of DRG cells. Scale bar: 50 µm. O,P) Representative silver staining (O) and GST pull‐down assay (P) of CD109 combined with the TRPV1 protein in DRG cells. Q) Co‐IP confirmed the interaction between CD109 and TRPV1. R) Representative PLA images showing the colocalization of CD109 and TRPV1 in the DRG membrane. Scale bar: 50 µm. S) Representative IF and quantification of PANC‐1‐EV_CD109_‐treated DRG cells with or without TRPV1 knockdown. Scale bar: 50 µm. Statistical significance was assessed using a 2‐tailed Student's t test, as shown in Figure A (right panel), and one‐way ANOVA followed by Dunnett's test, as shown in Figures E‐F, H, J‐K, M, and S (right panel). The data are presented as the means ± SDs of three independent experiments. *P < 0.05, **P < 0.01.

To evaluate the recipient cells of EV‐packaged circPNIT derived from *KRAS^G12D^* PDAC tumors in the tumor microenvironment (TME), EVs were labeled with PKH67 and co‐cultured with different stromal cells. Flow cytometry confirmed that compared to other stromal cells, PKH67‐labeled EVs were mainly taken up by the DRG in the TME (**Figures** **5**A and S7I, Supporting Information). However, circPNIT‐KO in *KRAS^G12D^* PDAC cells abolished the internalization of the corresponding EVs in the DRG (Figures 5B and S8A–C, Supporting Information). Accordingly, the fluorescence intensity of DRG cells incubated with PKH67‐labeled EVs was significantly higher than that of other stromal cells, and the internalization of EVs in DRG cells was significantly decreased in *KRAS^G12D^* PDAC with circPNIT‐KO (Figures 5B and S8D, Supporting Information).

EVs’ surface proteins serve as recognition signals, enabling their targeted transmission to specific cells.^[^ ^5^, ^16^ ^]^ Herein, we found that CD109 was located on the surface of *KRAS^G12D^* PDAC‐derived EVs, whereas Rab5B overexpression promoted the secretion of CD109^+^ EVs (Figure 5C). Moreover, using immunoelectron microscopy, we observed that circPNIT downregulation suppressed the enrichment of CD109 on the membrane of EVs derived from *KRAS^G12D^* PDAC cells (Figure 5D). This impaired the ability of these EVs to induce circPNIT expression in DRG cells. In contrast, PANC‐1‐EV_circPNIT_ and EVs derived from circPNIT‐overexpressing AsPC‐1 cells (AsPC‐1‐EV_circPNIT_) markedly upregulated circPNIT expression in DRG cells (Figure 5E,F). To confirm whether CD109 is involved in the targeting of *KRAS^G12D^* PDAC‐derived EVs to DRG cells, we blocked CD109 with a neutralizing antibody and demonstrated that the overexpression of circPNIT promoted the internalization of *KRAS^G12D^* PDAC‐derived EVs and their packaged circPNIT by DRG cells, whereas the blockade of CD109 significantly inhibited the delivery of EV‐packaged circPNIT to DRG cells (Figures 5G–K and S8E, Supporting Information). Next, we verified neural targeting of CD109 in an orthotopic xenograft model in vivo. Similarly, compared to the PANC‐1‐EV_vector_, PANC‐1‐EV_circPNIT_ was significantly enriched in the nerves of primary tumor tissue, and the CD109 neutralizing antibody effectively inhibited the accumulation of EV in nerves (Figure 5L,M), indicating that CD109 mediated the targeted delivery of *KRAS^G12D^* PDAC‐derived EVs to neurons.

As the targeting specificity of EVs to cells is achieved by the specific binding of EV surface proteins to cell membrane receptors,^[^ ^17^ ^]^ we further explored the receptors recognized by CD109 on the surface of DRG cells. Confocal microscopy confirmed that the GST‐labeled recombinant CD109 protein was localized on the surface of the DRG cell membrane (Figure 5N). GST pull‐down assays confirmed an evident band in the 71–117 kDa range, which was identified as TRPV1 (Figure 5O,P). Co‐IP assays confirmed the interaction between CD109 and TRPV1 (Figure 5Q). Moreover, CD109 and TRPV1 co‐localized on the membrane of DRG cells, while TRPV1 knockdown in DRG cells inhibited the internalization of *KRAS^G12D^* PDAC‐derived EVs (Figure 5R,S and S9A, Supporting Information). Taken together, these results demonstrated that *KRAS^G12D^* PDAC‐derived CD109^+^ EVs achieved targeted delivery of circPNIT to neurons by recognizing TRPV1 on the membrane surface.

### 2.8 EV‐Packaged circPNIT Upregulates DSCAML1 Expression in Neurons to Promote Axonogenesis

**Figure 6:**
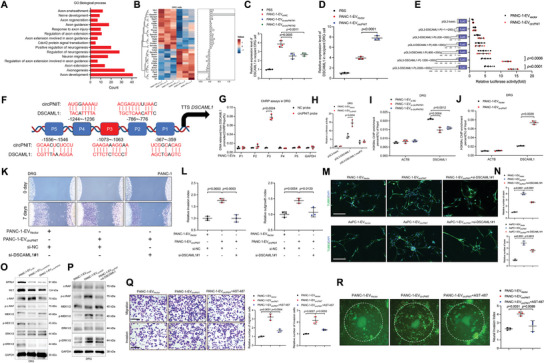
CD109^+^ EV‐packaged circPNIT induced axonogenesis to foster PNI. A) Axonogenesis associated biological process enrichment of differentially expressed genes identified from three pairs of DRG cells treated with PANC‐1‐EV_Vector_ or PANC‐1‐EV_circPNIT_. B) Heatmap of differentially expressed genes enriched in the GO biological process category associated with axonogenesis. C‐D) qRT‒PCR analysis of DSCAML1 expression in DRG cells treated with the indicated EVs. E) Transcriptional activity of DSCAML1 in DRG cells treated with truncated DSCAML1 promoter luciferase plasmids and EVs derived from PANC‐1 cells. F) Schematic representation of the predicted circPNIT binding sites in the DSCAML1 promoter. G) ChIRP assays detected circPNIT‐associated chromatin fragments in the DSCAML1 promoter in DRG cells. H) Luciferase activity in DRG cells treated with DSCAML1‐P3 promoter mutant luciferase plasmids and EVs derived from PANC‐1 cells. I‐J) ChIPChr‒qPCR assay of H3K9Ac enrichment on the DSCAML1 promoter in DRG cells treated with EVs derived from PANC‐1 cells as indicated. K‐L) Representative Matrigel/DRG images K) and quantification L) of neural invasion and outgrowth in PANC‐1 cells treated with the indicated EVs. Scale bar: 50 µm. M‐N) Representative images (M) and quantification (N) of the nerve density of DRG cells treated with EVs secreted by PANC‐1 and AsPC‐1 cells as indicated. O‐P) Western blotting of crucial proteins in the GFRα1/RET and MAPK/ERK signaling pathways in DRG cells treated with EVs derived from PANC‐1 cells as indicated. Q) Representative transwell images and quantification of the migration and invasion of PANC‐1 cells treated with EVs. Scale bar: 50 µm. R) Representative DRG‐matrix images and quantification of neural invasion by PANC‐1 cells treated with the indicated EVs and the GFRα1/RET inhibitor AST‐487. Scale bar: 50 µm. Statistical significance was assessed using a 2‐tailed Student's t test (Figures E, G, H, and J). One‐way ANOVA followed by Dunnett's test was used in Figures C‐D, I, L, N, and Q‐R(right panel). The data are presented as the mean ± SD of three independent experiments. *P < 0.05, **P < 0.01.

We further explored the mechanism by which *KRAS^G12D^* PDAC EV‐packaged circPNIT regulates axonogenesis after internalization into DRG cells. NGS analysis was performed on three pairs of independent DRG cells induced by PANC‐1‐EV_circPNIT_ or PANC‐1‐EV_Vector_. Gene Ontology analysis revealed enrichment of the axonogenesis pathway in DRG cells after PANC‐1‐EV_circPNIT_ incubation, and 32 genes that were differentially expressed in these pathways were enriched (**Figure** **6**A,B and S8F,G, Supporting Information). Subsequently, using qRT‐PCR, we found that DSCAML1 was the most upregulated gene in EV‐packaged circPNITs (Figure 6C,D and S8H,I, Supporting Information). DSCAML1 is a crucial regulator of neural development that participates in the growth and branching of neuronal axons.^[^ ^18^ ^]^ To further explore the mechanisms of EV‐packaged circPNIT‐induced DSCAML1 overexpression in DRG cells, a series of truncated sequences of DSCAML1 promoters spanning from ‐2000 to +200 bp relative to the transcriptional start site were cloned and inserted into luciferase reporter genes. Dual‐luciferase assays revealed that EV‐packaged circPNIT promoted transcriptional activity when DRG cells were transfected with the ‐800 to ‐1200 bp region of the DSCAML1 promoter (Figures 6E and S8J, Supporting Information). Moreover, Chromatin Isolation by RNA Purification assays confirmed that EV‐packaged circPNIT directly interacted with ‐1063 to ‐1073 bp of the DSCAML1 promoter (referred to as DSCAML1‐P3) in DRG cells (Figures 6F,G and S8K, Supporting Information). Mutation of the DSCAML1‐P3 region reduced the luciferase activity induced by EV‐packaged circPNIT (Figures 6H and S8L, Supporting Information), indicating that DSCAML1‐P3 was crucial for EV‐packaged circPNIT‐induced DSCAML1 upregulation in DRG cells. Specific patterns of histone modification in promoter regions are hallmarks of transcriptional activation.^[^ ^19^ ^]^ We found that the enrichment of H3K9Ac at the DSCAML1 promoter was strongly associated with EV‐packaged circPNIT expression (Figures 6I,J and S8M,N, Supporting Information), indicating that EV‐packaged circPNIT increased H3K9Ac levels at the DSCAML1 promoter to drive its transcriptional activation.

Next, we assessed whether DSCAML1 was indispensable for EV‐packaged circPNIT‐induced axonogenesis. The Matrigel/DRG model revealed that EV‐packaged circPNIT overexpression enhanced the migration and extension of neural axons toward PDAC cells, which was impaired by the downregulation of DSCAML1 (Figure 6K,L). Moreover, incubation with EV‐packaged circPNIT alone promoted axonal outgrowth of neurons, whereas blocking DSCAML1 reversed EV‐packaged circPNIT‐induced axonogenesis (Figure 6M,N). Collectively, these results suggested that EV‐packaged circPNIT promoted the transcriptional activation of DSCAML1 by recruiting H3K9Ac to the ‐1063 to ‐1073 bp region of DSCAML1, thereby promoting axonogenesis in *KRAS^G12D^* PDAC cells.

### 2.9 EV‐Packaged circPNIT‐Induced Axonogenesis Facilitates PNI by Activating GFRα1/RET Signaling

Next, we explored how EV‐packaged circPNIT‐induced axonogenesis promotes PNI in *KRAS^G12D^* PDAC tumors. Extension of axons is reportedly accompanied by the expression of receptors that promote cancer‐neuron crosstalk to achieve local invasion and metastasis.^[^ ^3^, ^20^ ^]^ Thus, the expression levels of core molecules involved in PNI‐related pathways in neurons were evaluated, and the results showed that the expression of only GFRα1 and RET was positively correlated with the expression of EV‐packaged circPNIT in DRG cells (Figure S8O, Supporting Information). Given that the GFRα1/RET complex is considered the major pathway leading to neural tracking and adhesion via downstream MAPK/ERK signaling during PNI,^[^ ^20^, ^21^ ^]^ we further assessed the activation of the GFRα1/RET pathway. Western blot assays showed that knockdown of EV‐packaged circPNIT significantly decreased GFRα1 and RET expression and reduced the phosphorylation of c‐RAF, MEK1/2, and ERK1/2, while overexpressing EV‐packaged circPNIT significantly promoted GFRα1 and RET expression and c‐RAF, MEK1/2, and ERK1/2 phosphorylation (Figures 6O,P and S8P,Q, Supporting Information). Moreover, downregulation of DSCAML1 reversed the increase in GFRα1 and RET expression and MAPK/ERK pathway activation induced by EV‐packaged circPNIT (Figures 6P and S8Q, Supporting Information), suggesting that EV‐packaged circPNIT activated the GFRα1/RET pathway in DRG cells by triggering DSCAML1‐induced axonogenesis.

To further elucidate whether GFRα1/RET pathway activation is essential for EV‐packaged circPNIT‐induced PNI in *KRAS^G12D^* PDAC, transwell assays were performed, which revealed that DRG cells precultured with EV‐packaged circPNIT significantly promoted the invasion of *KRAS^G12D^* PDAC cells into DRG cells, while treatment with the RET inhibitor AST‐487 reversed this effect (Figure 6Q). Moreover, a DRG‐matrix assay further confirmed that overexpression of EV‐packaged circPNIT promoted the neurotrophic ability of *KRAS^G12D^* PDAC cells, whereas AST‐487 inhibited EV‐packaged circPNIT‐induced neurotropism in *KRAS^G12D^* PDAC cells (Figure 6R). In conclusion, EV‐packaged circPNIT‐induced axonogenesis promotes the PNI of *KRAS^G12D^* PDAC by activating the GFRα1/RET pathway.

### 2.10 CD109^+^EV‐Mediated Targeted Delivery of circPNIT Promotes Axonogenesis and PNI of KRAS^G12D^ PDAC in Mice

**Figure 7:**
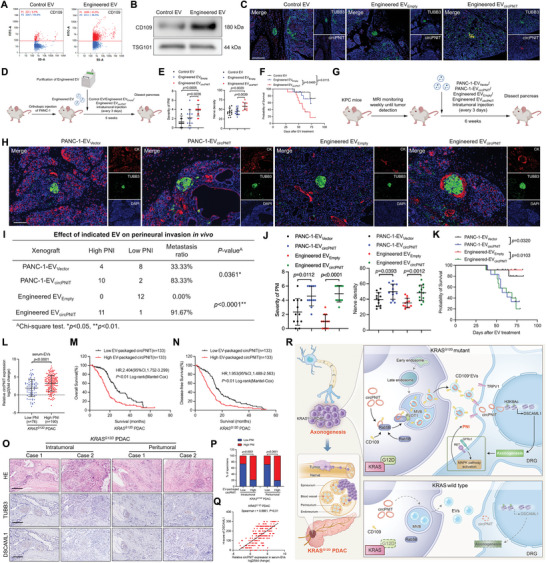
CD109^+^EVs‐mediated targeted delivery of circPNIT promotes axonogenesis and PNI of *KRAS^G12D^* PDAC in mice. A,B) The nanoflow cytometric analysis (A) and western blotting (B) were used to evaluate the expression of CD109 in Engineered EV. C) Representative images the circPNIT targeting engagement in the PANC‐1 induced tumor tissue. Scale bar: 5 µm. D) Schematic illustration of the establishment of the EV‐induced orthotopic xenograft model. E) Quantification of the PNI severity and nerve density in the primary tumor tissues treated with indicated EVs. F) The survival times of the mice treated with indicated EVs. G) Schematic illustration of the KPC spontaneous tumorigenesis mouse model. H‐I) Representative mIHC images (H) and quantification of neural invasion (I). Scale bar: 50 µm. One‐way ANOVA followed by Dunnett's test was used. J) Quantification of PNI severity and nerve density in KPC mouse tumor tissue treated with indicated EVs. Scale bar: 50 µm. One‐way ANOVA followed by Dunnett's test was used. K) Kaplan‐Meier analysis of survival time after treatment with indicated EVs. L) qRT‒PCR analysis of cirPNIT expression in serum EVs obtained from 266 *KRAS^G12D^* PDAC patients with high PNI or low PNI. The nonparametric Mann‒Whitney *U* test was used. M‐N) Kaplan‐Meier survival analysis of OS and DFS in patients with *KRAS^G12D^* PDAC according to the EV‐packaged circPNIT expression level (the cutoff value was the median). O‐P) Representative H&E and IHC images (O) and percentages (P) of PNI in *KRAS^G12D^* PDAC patients according to EV‐packaged circPNIT expression. Scale bars: 50 µm. The χ^2^ test was used to assess statistical significance. Q) Correlation analysis of DSCAML1 expression in tumor tissues and serum circPNIT levels in a cohort of 266 patients with *KRAS^G12D^* PDAC. R) The proposed model of how *KRAS^G12D^* PDAC‐secreted EV‐packaged circPNIT induces the DSCAML1/GFRα1/RET axis to promote axonogenesis and PNI in *KRAS^G12D^* PDAC. The data are presented as the mean ± SD of three independent experiments. *P < 0.05, **P < 0.01.

Given that *KRAS^G12D^* PDAC‐derived CD109^+^EVs deliver circPNIT to neurons and trigger axonogenesis‐induced PNI, we sought to determine the targeted engagement of circPNIT in the primary tumor tissue generated by *KRAS^G12D^* PDAC. To achieve the targeted engagement of circPNIT in neurons, we constructed engineered EVs to simulate neuron‐targeted CD109^+^EVs derived from *KRAS^G12D^* PDAC cells, which were confirmed to be loaded with CD109 on the surface through nanoflow cytometry and western blot analyses (**Figure** **7**A,B). Moreover, fluorescence staining confirmed that engineered EV_circPNIT_ enhanced spot fluorescence in nerves in an orthotopic xenograft model compared to that in control EV and engineered EV_Empty_ (Figure 7C,D), suggesting that engineered EV_circPNIT_ has significant neuronal targeting and delivers circPNIT to nerves in the primary tumor tissue. The PNI score showed that engineered EV containing circPNIT, compared with control EV and engineered EV_Empty_, promoted the severity of PNI and nerve density in the primary tumor tissue constructed by PANC‐1 (Figure 7E). Meanwhile, the survival time of engineered EV_circPNIT_‐treated nude mice was significantly shorter than those of the control and engineered EV_Empty_ (Figure 7F).

Further validation was performed using the KPC model to explore the PNI effect of engineered EV‐packaged circPNIT in *KRAS^G12D^* mutation‐induced tumor tissues. Once the primary tumor was detected, PANC‐1‐EV_Vector_, PANC‐1‐EV_circPNIT_, engineered EV_Empty_, and engineered EV_circPNIT_ were injected into the pancreas every 3 days for 6 weeks (Figure 7G). The PNI score showed that PANC‐1‐EV‐loaded circPNIT or engineered EV‐packaged circPNIT exacerbated the severity of PNI in mouse tumor tissue compared to that in the control group, indicating that circPNIT triggers KRAS^G12D^ PDAC‐derived EV to induce PNI (Figure 7H–K). Conversely, engineered EV‐loaded si‐circPNIT (loading efficiency: 3.06% ± 0.48%) significantly reduced PNI severity and nerve density in comparison to those of control EV and engineered EV_si‐NC_ (Figure S9B–E, Supporting Information). Moreover, survival time after EV treatment was longer in the engineered EV_si‐circPNIT_ group than in the control group (Figure S9F, Supporting Information). Taken together, these results indicate that *KRAS^G12D^* PDAC‐derived CD109^+^EV promote PNI in *KRAS^G12D^* mutant tumor tissues by transferring circPNIT.

### 2.11 Clinical Relevance of EV‐Packaged circPNIT in KRAS^G12D^ PDAC

We further evaluated the clinical relevance of EV‐packaged circPNIT in *KRAS^G12D^* PDAC. The expression of serum EV circPNIT in *KRAS^G12D^* PDAC patients with high PNI was significantly higher than that in patients with low PNI, indicating a positive correlation between serum EV circPNIT and the PNI of *KRAS^G12D^* PDAC (Figure 7L). Moreover, Kaplan–Meier analysis revealed that *KRAS^G12D^* PDAC patients with higher serum EV circPNIT expression had shorter OS and DFS rates (Figure 7M,N). Univariate and multivariate analyses revealed that higher circPNIT expression was an independent predictor of poor survival of *KRAS^G12D^* PDAC patients (Tables S2 and S3, Supporting Information). Furthermore, IHC analysis revealed that patients with upregulated serum EV circPNIT expression had elevated DSCAML1 levels, which were associated with increased PNI severity (Figure 7O–Q). In conclusion, our results indicated that serum EV circPNIT has potential diagnostic and predictive value for neuroinvasive *KRAS^G12D^* PDAC (Figure 7R).

## 3 Discussion

PDAC with *KRAS* mutations is accompanied by nerve infiltration in the early stages of tumor progression, which is a core driving force for postoperative recurrence and poor prognosis.^[^ ^22^ ^]^ However, the mechanisms underlying the PNI in *KRAS*‐mutant PDAC remain unclear. Herein, we reported for the first time that the *KRAS^G12D^* mutation was positively associated with a high PNI in PDAC patients via a multicenter analysis. We identified *KRAS^G12D^* PDAC derived CD109^+^ EVs that transmit circPNIT to foster tumor‐associated axonogenesis and PNI, both in vitro and in vivo. In *KRAS^G12D^* PDAC, circPNIT formed a ternary complex by binding to Rab5B and its effector—CD109. Subsequently, CD109 directed Rab5B anchoring to lipid rafts on MVBs, bypassing the traditional endosomal trafficking pathway and facilitating circPNIT secretion as a cargo of CD109^+^ EVs in a lipid raft‐dependent manner. Moreover, we showed that *KRAS^G12D^* PDAC‐derived CD109^+^ EVs delivered circPNIT to neurons by recognizing TRPV1 on the neuronal membrane, thus triggering axonogenesis induced by DSCAML1 activation. Next, neurons accompanied by axonogenesis expressed high levels of GFRα1/RET, which in turn enhanced the neurotropism of PDAC. Our study highlighted the mechanism bridging *KRAS* mutation‐mediated EVs and PNI and identified CD109^+^ EV‐packaged circPNIT as an effective target for early intervention in PDAC patients with PNI.

Although progress has been made in the diagnosis and treatment of PDAC, PNI remains the leading cause of disease recurrence and poor survival among these patients.^[^ ^23^ ^]^ Surgical strategies based on nerve ablation have a profound impact on controlling tumor progression, including delaying tumor recurrence and metastasis.^[^ ^24^ ^]^ Therefore, exploring the regulatory mechanism of PNI in PDAC has great clinical value for improving the prognosis of *KRAS^G12D^* PDAC. In this study, we identified tumor‐associated axonogenesis as the key determinant of PNI in *KRAS^G12D^* PDAC and demonstrated that axonogenesis is driven by the transcriptional activation of DSCAML1 in neurons, which is regulated by EV‐packaged circPNIT from *KRAS^G12D^*. Moreover, DSCAML1‐induced axonogenesis of neurons was accompanied by elevated expression of GFRα1/RET, the critical receptor mediating PNI. Moreover, blockage of DSCAML1 effectively suppressed *KRAS^G12D^* PDAC‐derived EV‐packaged circPNIT‐induced axonogenesis and PNI, implying the potential clinical utility of DSCAML1 as a therapeutic target for preventing neural invasion of PDAC.

Traditionally, the production of EVs relies on multistep transport through the endosomal system via Rab GTPases and ESCRT complexes, which are highly energy‐intensive processes.^[^ ^25^ ^]^ Dysfunction of the Rab GTPase‐mediated endosomal trafficking pathway leads to altered endocytosis and secretion of EVs.^[^ ^26^ ^]^ However, the regulatory mechanisms driving EV secretion in *KRAS^G12D^* PDAC have not yet been elucidated. Herein, we demonstrated an alternative mechanism that bypasses the traditional endosomal trafficking pathway involving Rab5B, thereby providing an efficient and energy‐saving pathway for EVs production in *KRAS^G12D^* PDAC. By specifically recognizing effectors, Rab5B directly localized to lipid rafts on MVBs under the guidance of CD109, thereby bypassing the conventional trafficking pathways of early and late endosomes. Our findings reveal a novel mechanism of EV production involving Rab5B in *KRAS^G12D^* PDAC that bypasses traditional endosomal transport, expanding our knowledge of the regulatory process by which PDAC cells adopt a more energy‐efficient mode of metastasis.

EVs have shown extensive application prospects in tumor therapy.^[^ ^27^ ^]^ Engineered EVs, which are essential carriers for tumor therapy, have shown satisfactory efficacy in treating a variety of tumors.^[^ ^28^ ^]^ This study showed that CD109^+^ EVs exhibited strong neuro‐targeting potential and are expected to constitute a breakthrough in the development of targeted therapies for neuroinvasive PDAC. Notably, CD109^+^ EVs harboring si‐circPNIT effectively inhibited axonogenesis and PNI in mouse models of spontaneous tumorigenesis, thus providing a feasible strategy for PNI treatment in *KRAS^G12D^* PDAC. Radical surgery in conjunction with chemotherapy is the prevailing therapeutic approach for PDAC. However, chemotherapy resistance is an unavoidable consequence of prolonged treatment in PDAC patients and is highly correlated with the PNI. This therapeutic strategy of targeting nerves is expected to improve chemoresistance and provide an adjuvant therapeutic option for comprehensive treatment of pancreatic cancer. Our study highlighted that CD109^+^EVs are essential for targeting the PNI of *KRAS^G12D^* PDAC, providing a modifiable tool for treating PDAC nerve infiltration.

Currently, validated assessment measures for PNI diagnosis in PDAC patients are lacking. Imaging modalities, such as CT and MR, cannot detect the occurrence of PNI. Therefore, the diagnosis of nerve metastases in PDAC patients remains challenging. In recent years, serum EVs have been widely used as an important measure in liquid biopsy for dynamic tumor detection and response assessment.^[^ ^29^ ^]^ In this study, we found that circPNIT was upregulated in the serum EVs of PDAC patients with severe PNI and that the detection of serum EV‐circPNIT can effectively differentiate PDAC patients with PNI, thus filling the gap in the diagnosis of PDAC neuroinfiltration.

In summary, this study revealed a bypass mechanism via the circPNIT/Rab5B/CD109 complex, distinct from conventional endosomal trafficking, highlighting the critical role of *KRAS^G12D^* PDAC‐derived EV‐packaged circPNIT in PNI. Moreover, we propose that CD109^+^ EVs, which effectively target neurons and promote the occurrence of PNI in *KRAS^G12D^* PDAC via circPNIT delivery, are attractive drug delivery carriers for the treatment of PNI.

## 4 Experimental Section

### Patients and Specimens

Data were collected from 530 PDAC patients who underwent pancreatic surgery at Guangdong Provincial People's Hospital, Sun Yat‐sen Memorial Hospital, and Sixth Affiliated Hospital, Sun Yat‐sen University. All patients were fully informed about and consented to participate in the study. PNI was diagnosed by two independent pathologists in a blinded manner. Collected PDAC tissues and paired NATs were stored in −80 °C for RNA or protein extraction and in formalin for mIHC), immunohistochemistry IHC, and in situ hybridization (ISH). Corresponding peripheral serum samples from PDAC patients and matched healthy volunteers were collected for subsequent experiments.

The PNI score was used to assess the severity, distribution, and frequency of PNI. Based on the location of PNI occurrence, it was categorized as 0‐no PNI 1‐PNI in the peritumoral region and 2‐PNI in the intratumoral region. Based on the frequency of PNI, it can be classified as 0‐no PNI, 1‐low, 2‐frequent, and 3‐excessive. The two scores were multiplied to calculate the severity of PNI in PDAC. The score < 4 was considered the “low PNI” group and the score ≥ 4 was considered the “high PNI” group.^[^ ^8^, ^30^ ^]^ Nerve density was quantified as described previously.^[^ ^31^ ^]^

### Cell Lines and Cell Culture

Human PDAC cell lines (*KRAS^G12D^*: PANC‐1, AsPC‐1, *KRAS^G12V^*: Capan‐2, *KRAS^G12C^*: MiaPaCa‐2, *KRAS^WT^*: BxPC‐3) and Human Umbilical Vein Endothelial Cells (HUVECs) were purchased from the American Type Culture Collection (ATCC, USA). Primary cancer‐associated fibroblasts (CAFs) and tumor‐associated macrophages (TAMs) were isolated from pancreatic cancer tissues using a Human Tumor Dissociation Kit (130‐095‐929, Miltenyi Biotec, Germany) following the manufacturer's instructions. T and B cells were isolated from the peripheral blood of healthy volunteers and cultured in RPMI 1640 medium (Invitrogen, USA). Human lymphatic endothelial cells (HLECs) were obtained from ScienCell Research Laboratories (Carlsbad, CA, USA, Cat#2500). PANC‐1, HUVECs, CAFs, and MiaPaCa‐2 cells were cultured in DMEM (Invitrogen, USA). Moreover, AsPC‐1, TAMs, and BxPC‐3 cells were cultured in RPMI 1640 (Invitrogen, USA). HLECs were cultured in an endothelial cell medium (ECM; ScienCell Research Laboratories, Cat#1001). Capan‐2 cells were cultured in McCoy's 5A medium (Invitrogen). DRG were cultured in DMEM supplemented with 10% FBS. All cells were cultured in a humidified incubator with 5% CO_2_ at 37 °C, and the authentication and mycoplasma testing were qualified through STR DNA profiling.

### Matrigel/DRG Model

As previously reported, DRG explants and tumor co‐culture experiments were used to evaluate the paracrine effects of tumor cells on neurons.^[^ ^32^ ^]^ Nude mice (female, 4‐weeks‐old, 18–20 g) were purchased from Guangdong Experimental Animal Center. DRG or 1 × 10^5^ DRG cells were isolated from nude mice and seeded in six‐well plates containing 10 µL of Matrigel (BD Biosciences, NY, USA). Moreover, 1 × 10^5^ PDAC cells were resuspended in 10 µL of Matrigel, seeded 2 mm from the edge of the DRG, and cultured in DMEM with 10% FBS at 37 °C in 5% CO_2_. Next, 20 µg of EVs was added to the culture medium. Cancer cell and DRG migration were evaluated 7 days later, as previously reported.^[^ ^32a^ ^]^ The distance between PDAC cells and the DRG (c), the migration of PDAC cells (α), and the migration of DRG cells (β) were defined. The invasion index (α/c) and outgrowth index (β/c) were used to quantify the neurotropism of PDAC cells and nerve outgrowth, respectively.

### DRG‐Matrix Assay

Nude mice (female, 4‐weeks‐old, 18–20 g) were purchased from Guangdong Experimental Animal Center. The DRG matrix was established as previously described.^[^ ^33^ ^]^ The DRG of the nude mice was isolated and washed with pre‐cooled PBS. The cells were fixed in six‐well plates containing 2 µL of growth factor‐reducing Matrigel (BD Biosciences, NY, USA). After 3 days of culture in 5% CO_2_, 1 × 10^5^ GFP‐tagged PDAC cells were added, and tumor cell invasion into the DRG was observed 2–3 days later, as previously reported.^[^ ^34^ ^]^ The fluorescent signals of the tumor cells invading the Matrigel were quantified using ImageJ (ImageJ, RRID:SCR_003070).

### Orthotopic Xenograft Model

Nude mice (female, 4‐weeks‐old, 18–20 g) were purchased from Guangdong Experimental Animal Center. The mice were anesthetized via pentobarbital inhalation and placed in the right lateral decubitus position. The left ventrolateral side was incised to expose the pancreas. Subsequently, 1 × 10^6^ PDAC cells were injected into the pancreas of each mouse. Six weeks later, the primary tumors were collected. HE and mIHC analyses were performed to assess nerve density and severity of PNI within the tumor tissues.

### In Vivo Model of Neural Infiltration

Nude mice (female, 4‐weeks‐old, 18–20 g) were purchased from Guangdong Experimental Animal Center. GFP‐labelled PDAC cells (1 × 10^5^) were injected into the sciatic nerve. One week later, the mice were randomly divided into different groups (n = 6) and injected with PBS or 50 µg of EVs into the right sciatic nerve for every 3 days. Sciatic nerve function was monitored weekly after injection. Six weeks later, MRI was performed, and the sciatic nerves were collected for HE staining.

Seven days after cell inoculation, sciatic nerve function was assessed using a neurological function score^[^ ^35^ ^]^ and graded from 5 (normal) to 0 (complete paralysis) based on the hind limb response to active body extension. The grading system was as follows: 5: normal, symmetrical hind limbs, response to hand movement to pull the body; 4: transient abnormal response of the right hind limb to manual traction; 3: frequent abnormal response to hand movement and traction of the right hind limb; 2: persistent abnormal response of the right hind limb to hand movement and traction; 1: significant paralysis of the right hind limb; 0: inability to bear weight on the hind limbs or tumor diameter ≥ 20 mm.

### Genetically Engineered Models

*KRAS^G12D/+^ Trp53^R172H/+^ Pdx‐1‐Cre* (KPC) mice were purchased from Shanghai Model Biology. Pancreatic cancer formation in KPC mice was monitored weekly using MRI. Once tumors were detected, the mice were randomly divided into different groups (n = 12), followed by intratumoral injection with 50 µg EVs every 3 days. The primary tumor internal nerves were analyzed via HE and IHC staining at the end of the experiment.

### Construction of Engineered EV

The CD109 and LAMP2B CDSs were digested with BamHI and EcoRI and subsequently purified on a gel. The CDS of CD109‐LAMP2B was ligated into the eukaryotic expression vector pCDNA3.1(+)‐GNSTM‐HA using T4 DNA ligase (Vazyme, Nanjing, China). Next, 293T cells were transfected with engineered EVs. At 72 h after cell transfection, the cell supernatant was collected and EVs were collected through ultracentrifugation for subsequent experiments.

### Statistical Analyses

All the quantitative data were calculated as the means ± SD of at least three independent experiments. Kaplan–Meier analysis was performed to calculate the OS and DFS. Independent prognostic factors were determined using multivariate Cox proportional hazard models. Statistical analyses were performed based on the nature of the variables, assumptions of data distribution, and efficacy. Two‐tailed Student's t‐test or one‐way ANOVA was performed to evaluate parametric variables. Chi‐square test or Fisher's exact test was performed to evaluate nonparametric variables. The threshold for statistical significance was set at *P* <  0.05. All the statistical tests were performed with GraphPad Prism 9 (version 9.4.1; GraphPad Software, USA) and SPSS (version 26.0.0; IBM SPSS Statistics, USA).

### Study Approval

All 530 PDAC tissues and NAT samples were obtained from patients (311 males and 219 females) who underwent pancreatic surgery at Guangdong Provincial People's Hospital, Sun Yat‐sen Memorial Hospital, and The Sixth Affiliated Hospital of Sun Yat‐sen University. This study was approved by the Committees for Ethical Review of Research involving Guangdong Provincial People's Hospital, Sun Yat‐sen Memorial Hospital, and the Sixth Affiliated Hospital of Sun Yat‐sen University (approval no. 2016 (136), approval no. KY‐D‐2021‐127‐01, approval no. 2022ZSLYEC‐295). The animal study was approved by the Guangdong Provincial People's Hospital (approval no. KY‐Z‐2021‐211‐02).

## Conflict of Interest

The authors declare no conflict of interest.

## Author Contributions

D.Z., Y.Lu, Y.Li, and Z.F. contributed equally to this work. C.C., R.C., and Y.L. participated in the study design. D.Z., Y.L., Z.F., and M.Y. performed in vitro and in vivo experiments. Z.F. and Q.X. performed clinical data analysis. M.A., H.Z., and C.Y. performed immunofluorescence and mIHC experiments. Z.W. and J.Y. interpreted data and revised the manuscript. D.Z. and Y.L. wrote the manuscript. All the authors have read and approved the final version of the manuscript.

## Supporting information



Supporting Information

## Data Availability

All data in this study are presented in the article or supplementary material. The RNA sequencing data generated in this study are available in the Gene Expression Omnibus under accession numbers GSE234760 (https://www.ncbi.nlm.nih.gov/geo/query/acc.cgi?acc=GSE234760)andGSE251916 (https://www.ncbi.nlm.nih.gov/geo/query/acc.cgi?acc=GSE251916).
