# Supplementary material for: Endosomal Trafficking Bypassed by the RAB5B‐CD109 Interplay Promotes Axonogenesis in KRAS‐Mutant Pancreatic Cancer
Source: Adv Sci (Weinh). 2024 Nov 3;11(47):2405092. doi: 10.1002/advs.202405092 (PMC11653710; doi:10.1002/advs.202405092)
Supplement: Supplementary file 1 — Supporting Information [file ADVS-11-2405092-s001.docx]

**Supplemental Figures**

**
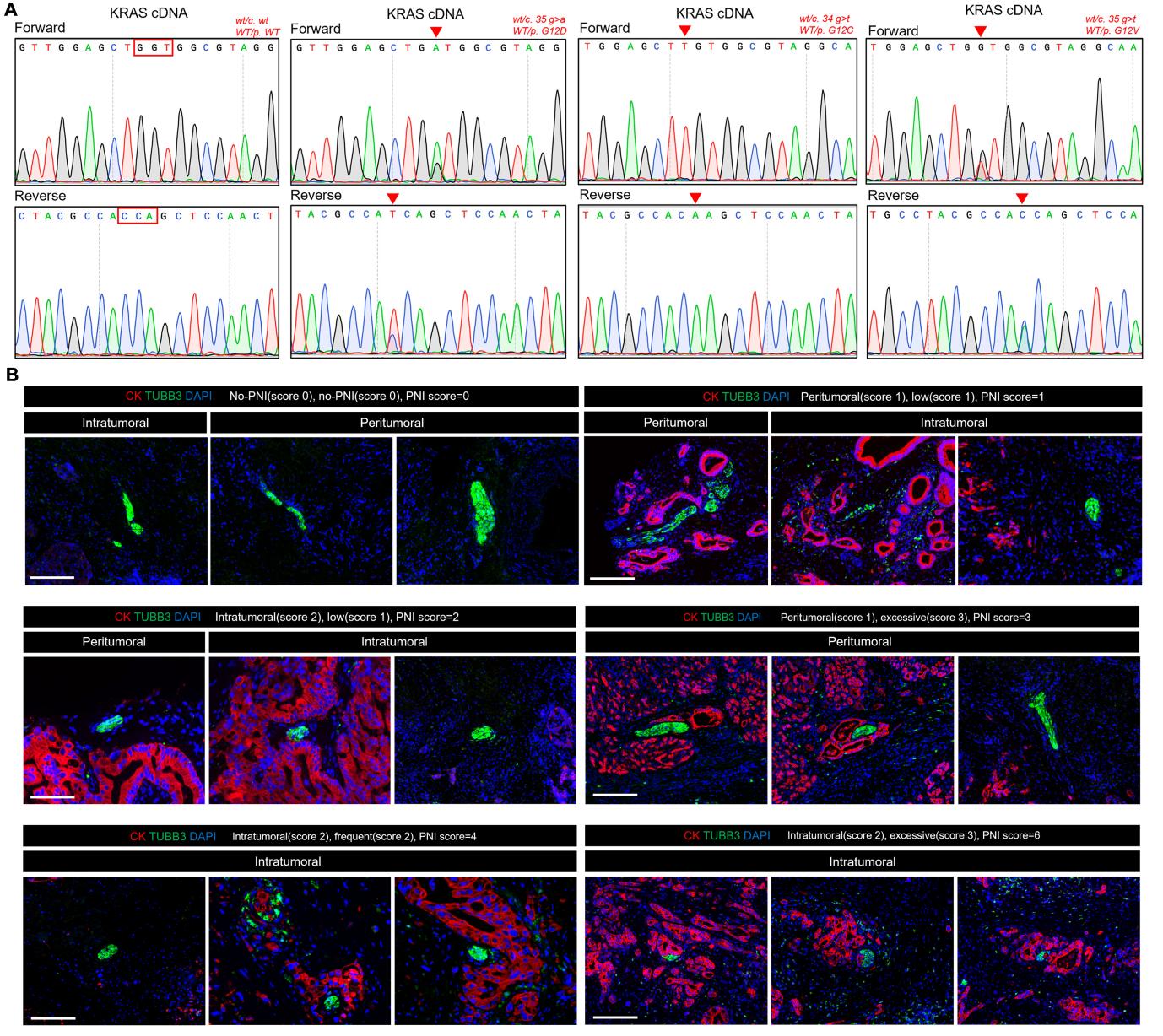
**

**Figure S1. The evaluation of PNI severity in KRAS mutant PDAC tumor tissue.** (**A**) *KRAS^WT^*, *KRAS^G12D^*, *KRAS^G12C^*, and *KRAS^G12V^* mutations were identified by sanger sequencing. (**B**) Representative images of PNI severity within KRAS-mutant PDAC tissue at different PNI scores. Scale bars: 50 μm.


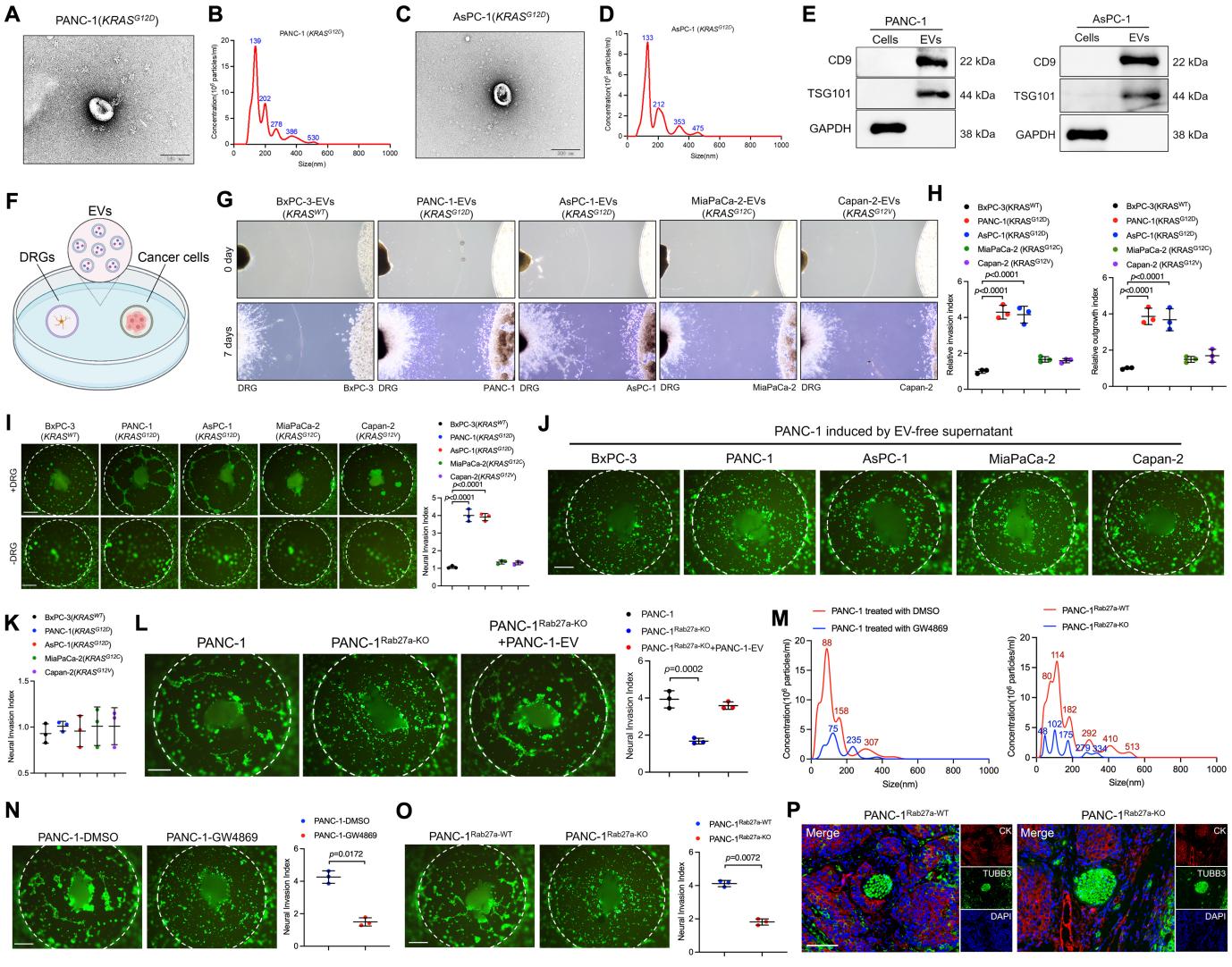


**Figure S2. *KRAS^G12D^* PDAC-derived EV-packaged circPNIT promoted axonogenesis and PNI.** (**A-E**) Purified PANC-1 and AsPC-1 EVs were identified by TEM, NTA, and Western blotting. Scale bars: 100 nm. (**F**) Schematic illustration of the Matrigel/DRG model of PDAC cells with the indicated *KRAS* subtypes and DRG cells. (**G-H**) Representative images (G) and quantification (H) of neural invasion and outgrowth in the Matrigel/DRG model of PDAC cells with the indicated *KRAS* subtypes treated with EVs. Scale bar: 50 μm. One-way ANOVA followed by Dunnett’s test was used. (**I**) Representative images and quantification of DRG matrix assay treated with the indicated *KRAS* mutant PDAC cells and corresponding EVs. Scale bar: 50 μm. One-way ANOVA followed by Dunnett’s test was used. (**J-K**) Representative images (J) and quantification (K) of DRG matrix assay treated with PANC-1 cells and *KRAS* mutant PDAC derived EV-free supernatant. Scale bar: 50 μm. One-way ANOVA followed by Dunnett’s test was used. (**L**) Representative images and quantification of DRG matrix assay treated with PANC-1 cells as indicated. Scale bar: 50 μm. One-way ANOVA followed by Dunnett’s test was used. (**M**) The NTA results of EVs derived from PANC-1 treated with GW4869 or Rab27a-knockout. (**N-O**) Representative images and quantification of DRG matrix assay treated with PANC-1 cells as indicated. Scale bar: 50 μm. One-way ANOVA followed by Dunnett’s test was used. (**P**) The orthotopic xenograft model constructed by PANC-1 treated with Rab27a-KO. Scale bars: 50 μm. Statistical significance was assessed using a 2-tailed Student’s t test, as shown in Figures N-O(right panel). One-way ANOVA followed by Dunnett’s test is shown in Figures H, I(right panel), K and L(right panel). The data are presented as the mean ± SD of three independent experiments. **P*<0.05, ***P*<0.01.


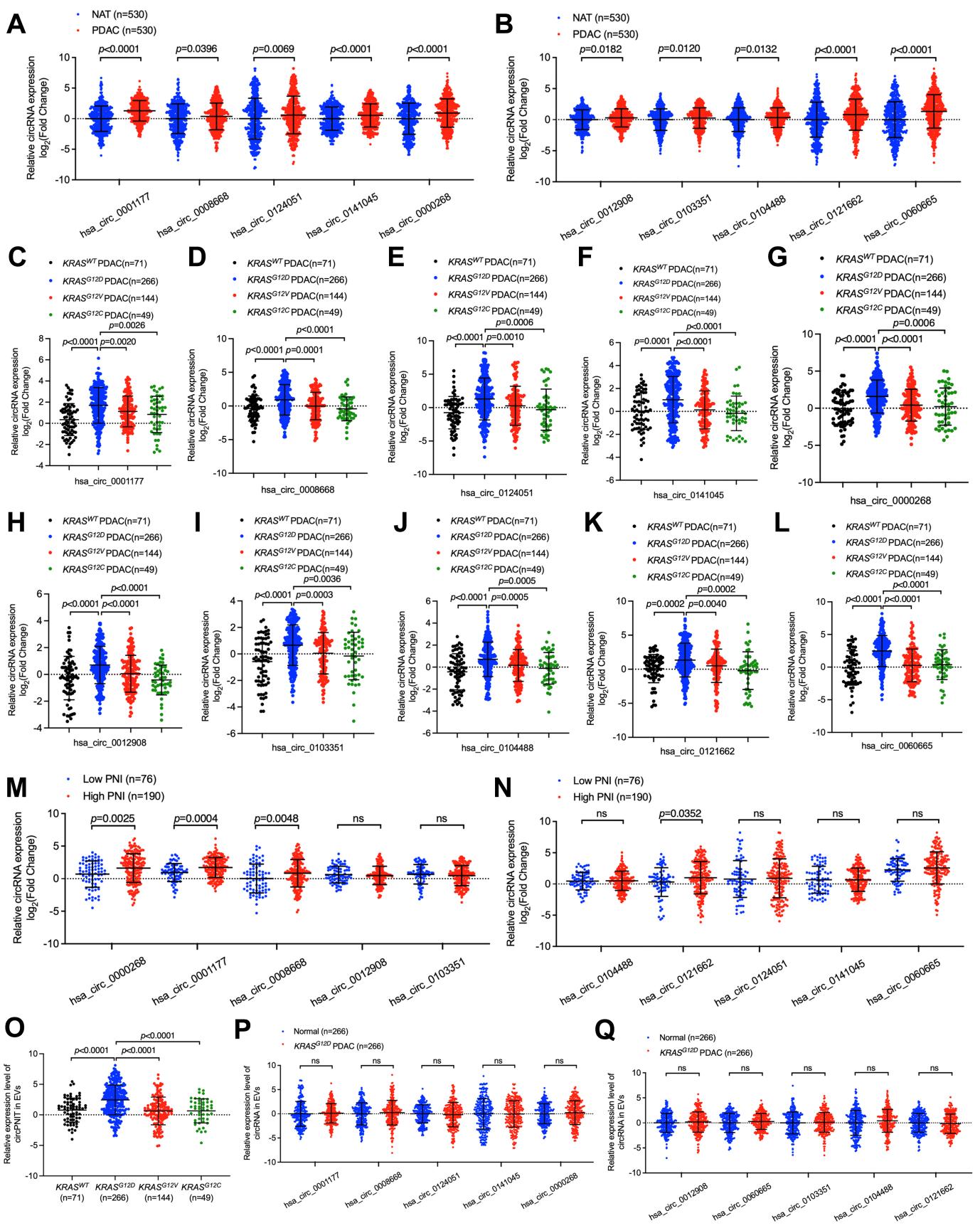


**Figure S3. The screening of critical circRNAs associated with *KRAS^G12D^* mutant PDAC.** (**A-B**) The results of 10 circRNA expression in 530-case PDAC tumor tissue and paired NAT. The nonparametric Mann‒Whitney *U* test was used. (**C-L**) The results of 10 circRNA expression in PDAC tissues with *KRAS* mutation. The nonparametric Mann‒Whitney *U* test was used. (**M-N**) The expression of 10 circRNAs in *KRAS^G12D^* PDAC tissues according to the PNI severity. The nonparametric Mann‒Whitney *U* test was used. (**O**) The expression of circPNIT in PDAC tissues with *KRAS* mutation. The nonparametric Mann‒Whitney *U* test was used. (**P-Q**) The expression of 10 circRNAs in serum EVs derived from *KRAS^G12D^* PDAC patients and paired normal volunteers. The nonparametric Mann‒Whitney *U* test was used. The data are presented as the mean ± SD. **P*<0.05, ***P*<0.01.


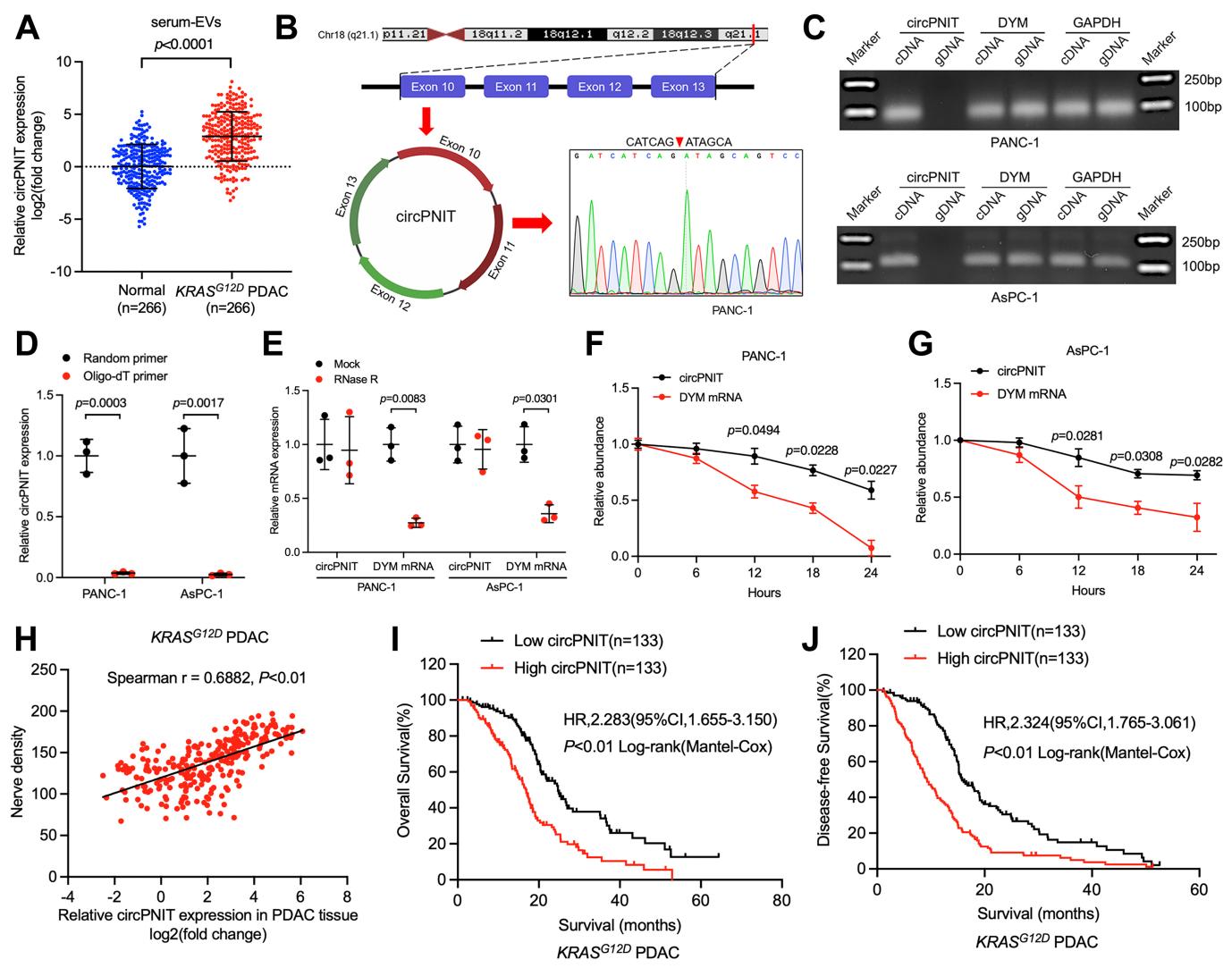


**Figure S4. circPNIT is upregulated in *KRAS^G12D^*-PDAC and associated with poor prognosis.** (**A**) qRT‒PCR analysis of circPNIT expression in serum EVs from 266 patients with *KRAS^G12D^* PDAC and 266 healthy participants. The nonparametric Mann‒Whitney *U* test was used. (**B**) Schematic illustration of the DYM gene locus and the back-spliced junction of circPNIT identified by Sanger sequencing. (**C**) PCR with agarose gel electrophoresis assays for circPNIT and DYM in the cDNA and gDNA of PANC-1 or AsPC-1 cells. (**D**) Relative circPNIT expression was evaluated by qPCR using random primers or oligo-dT primers. (**E**) circPNIT and DYM mRNA expression were analyzed by qPCR following RNase R treatment in PANC-1 and AsPC-1 cells. (**F-G**) Actinomycin D assays were used to assess the stability of circPNIT and DYM mRNA in PANC-1 (F) and AsPC-1 (G) cells at the indicated time points. (**H**) Correlation analysis of tumor tissue nerve density and circPNIT expression in a cohort of 266 patients with *KRAS^G12D^* PDAC. (**I-J**) Kaplan-Meier survival analysis of OS (I) and DFS (J) in patients with *KRAS^G12D^* PDAC according to the circPNIT expression level (the cutoff value is the median). Statistical significance was assessed using a 2-tailed Student’s t test, as shown in Figures D-G. The data are presented as the mean ± SD of three independent experiments. **P*<0.05, ***P*<0.01.


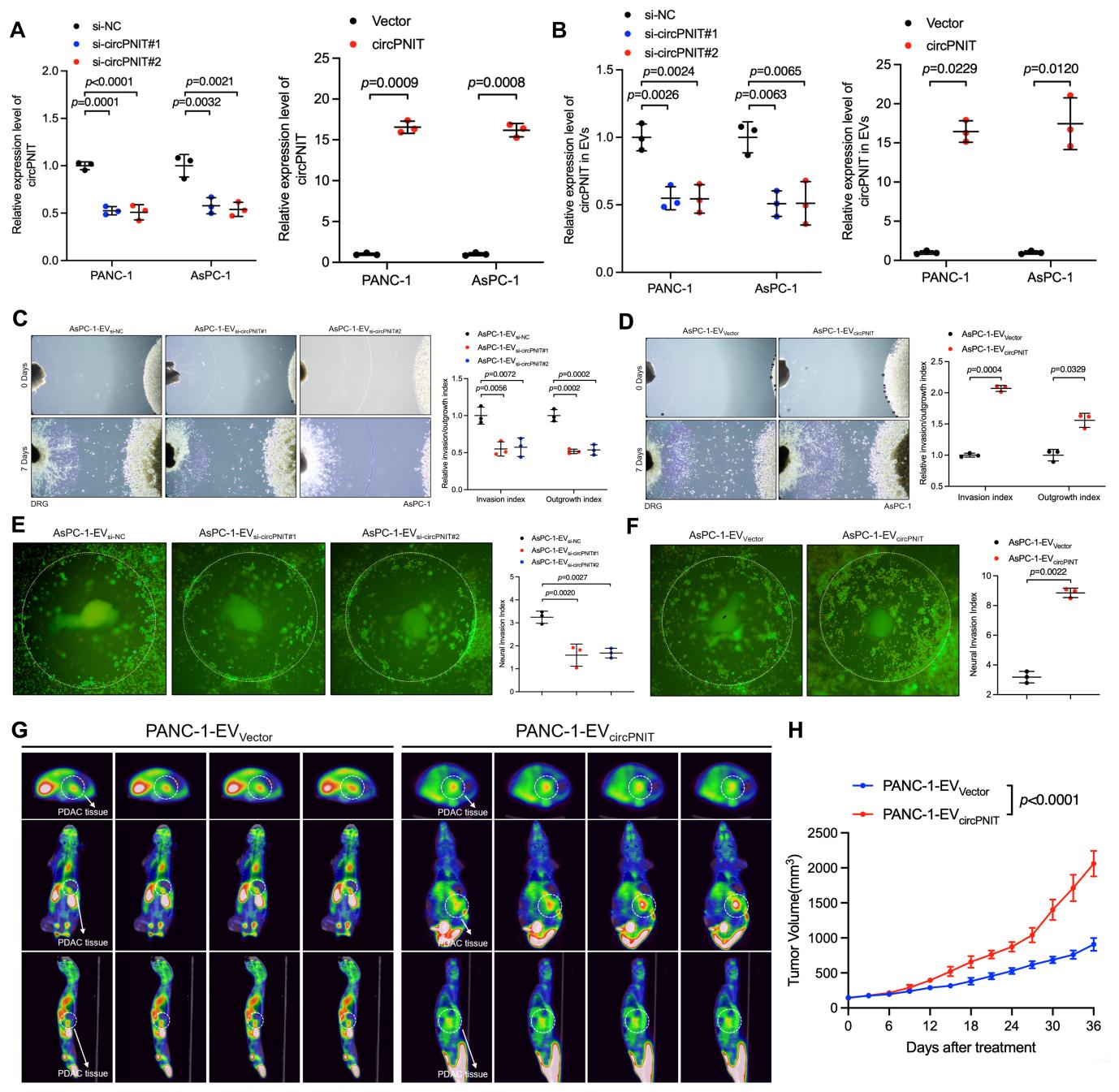


**Figure S5. *KRAS^G12D^* PDAC-derived EV-packaged circPNIT promoted axonogenesis and PNI.** (**A**) qRT‒PCR analysis of circPNIT expression in PANC-1 and AsPC-1 cells after circPNIT knockdown or overexpression. (**B**) qRT‒PCR analysis of circPNIT expression in EVs after circPNIT knockdown or overexpression. (**C-D**) Representative Matrigel/DRG images and quantification of neural invasion and outgrowth in AsPC-1 cells treated with the indicated EVs. Scale bar: 50 μm. (**E-F**) Representative DRG-matrix images and quantification of neural invasion by AsPC-1 cells treated with the indicated EVs. Scale bar: 50 μm. (**G**) The full perspective image of the PET-CT results in the orthotopic xenograft model. (**H**) The tumor volume of orthotopic xenograft model constructed by PANC-1 treated by indicated EVs. Statistical significance was assessed using a 2-tailed Student’s t test in Figures A-B (right panel), D (right panel),F (right panel) and H. One-way ANOVA followed by Dunnett’s test is shown in Figures A-B (left panel), C (right panel) and E (right panel). The data are presented as the mean ± SD of three independent experiments. **P*<0.05, ***P*<0.01.


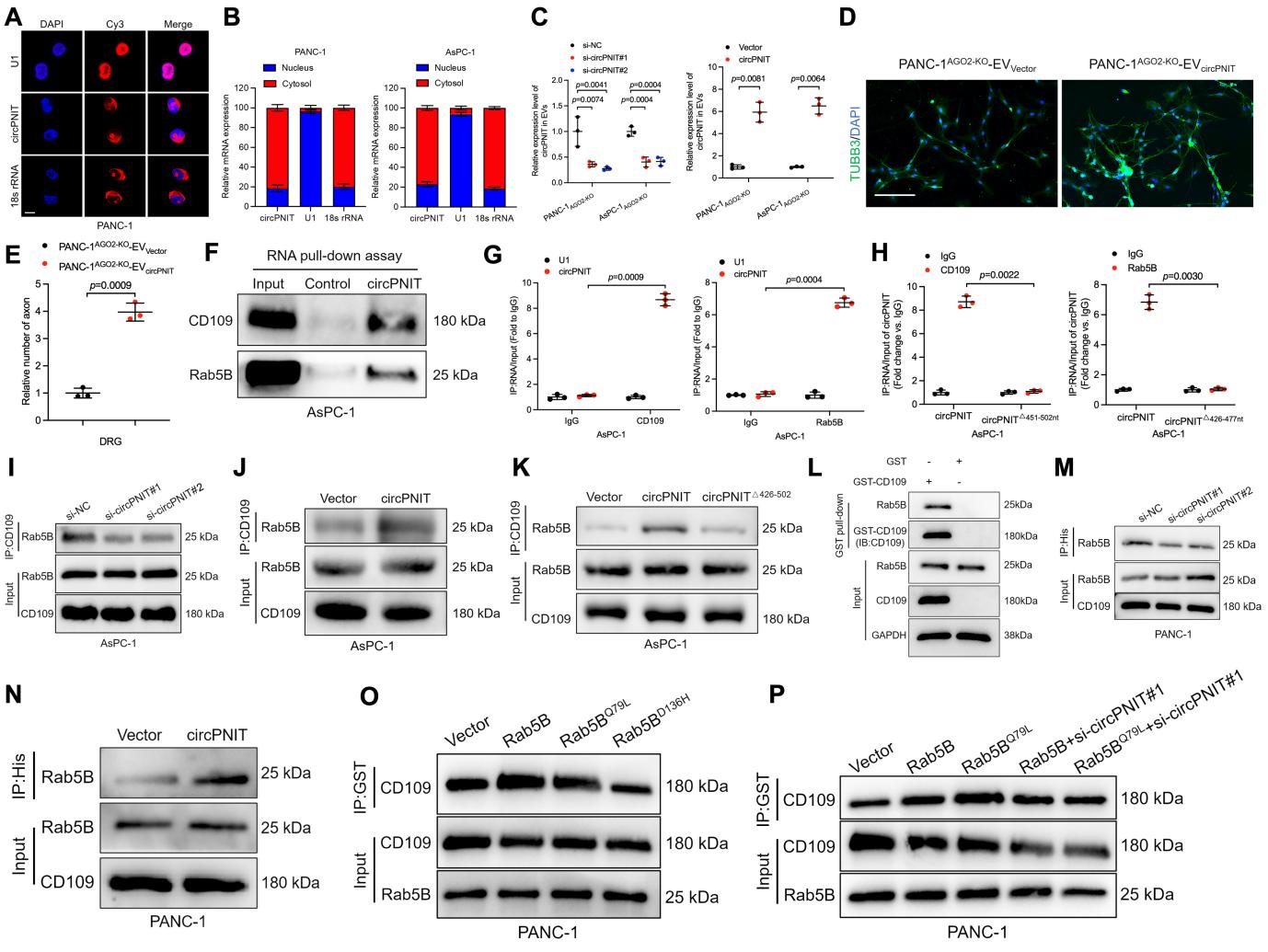


**Figure S6. The circPNIT/Rab5B/CD109 form a ternary complex.** (**A**) FISH assay of the subcellular distribution of circPNIT in PANC-1 cells. Scale bar: 10 μm. (**B**) Nuclear fractionation analysis and qRT‒PCR analysis of circPNIT expression in the nucleus and cytoplasm. Two-tailed Student’s t test and one-way ANOVA followed by Dunnett’s test were used. (**C**) qRT‒PCR analysis of circPNIT expression in EVs derived from AGO2-silenced *KRAS^G12D^* PDAC cells. Two-tailed Student’s t test and one-way ANOVA followed by Dunnett’s test were used. (**D-E**) Representative images (D) and quantification (E) of nerve density in DRG cells treated with AGO2-silenced PANC-1-derived EVs. Scale bar: 50 μm. A 2-tailed Student’s t test was used. (**F**) Pull-down and western blotting assays confirmed that circPNIT was associated with Rab5B and CD109 in AsPC-1 cells. (**G**) RIP assays confirmed that circPNIT interacted with CD109 and Rab5B in AsPC-1 cells. Negative control: IgG; nonspecific control: U1. Statistical significance was assessed using a 2-tailed Student’s t test. (**H**) RIP assays were performed after the mutation of circPNIT^451-502nt^ and circPNIT^426-477nt^ in AsPC-1 cells. A 2-tailed Student’s t test was used. (**I-J**) Co-IP assays analyzing the interaction between circPNIT-mediated CD109 and Rab5B in AsPC-1 cells. (**K**) Co-IP assays analyzing the interaction between CD109 and Rab5B after the circPNIT^426-502nt^ mutation in AsPC-1 cells. (**L-N**) GST pull-down and co-IP assay analyzing the interaction between circPNIT-mediated CD109 and Rab5B. (**O-P**) Co-IP assays analyzing the interaction between circPNIT-mediated CD109 and Rab5B were RabGTP dependent. The data are presented as the mean ± SD of three independent experiments. **P*<0.05, ***P*<0.01.


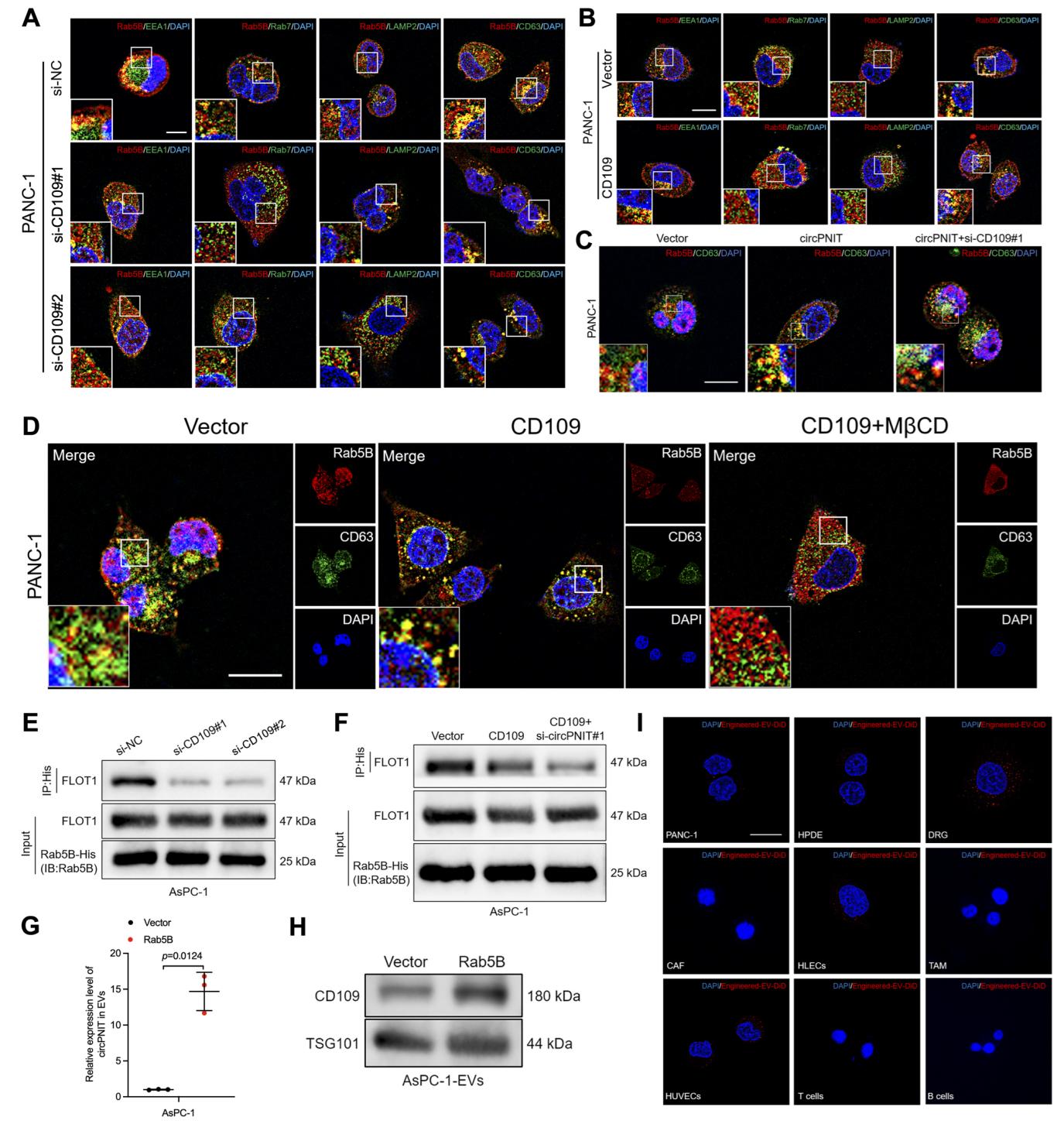


**Figure S7. The Rab5B-CD109 interaction bypasses endosomal localization to MVBs.** (**A-B**) Representative IF images showing the colocalization of Rab5B in the endosomal system mediated by knockdown (A) or overexpression of CD109 (B) in PANC-1 cells. Scale bar: 5 μm. (**C**) Representative IF images showing the colocalization of Rab5B and CD63 mediated by circPNIT overexpression or CD109 knockdown in PANC-1 cells. Scale bar: 5 μm. (**D**) Representative IF images showing the colocalization of Rab5B and CD63 in cells treated with a lipid raft inhibitor. Scale bar: 5 μm. (**E-F**) Co-IP and western blotting confirmed the interaction between Rab5B and FLOT1 in AsPC-1 cells. (**G**) qRT‒PCR analysis of circPNIT expression in EV derived from AsPC-1 cells treated with Rab5B overexpression lentivirus. (**H**) Western blotting analysis of the expression of CD109 in AsPC-1-derived EVs. (**I**) Representative IF images of stromal cells treated with DiD-labeled Engineered EV. Scale bar: 5 μm. Statistical significance was assessed using a 2-tailed Student’s t test in Figures G. The data are presented as the mean ± SD of three independent experiments. **P*<0.05, ***P*<0.01.


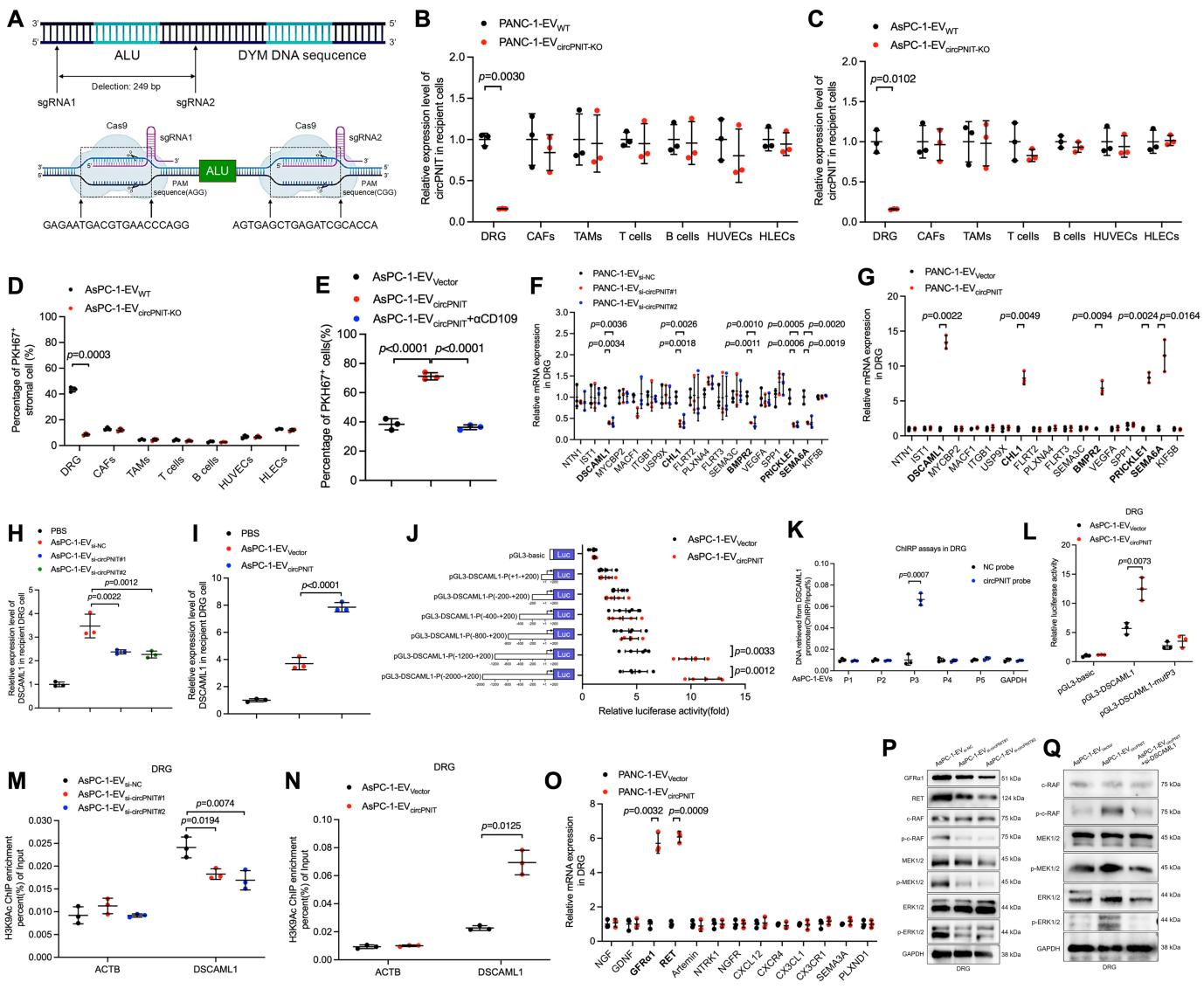


**Figure S8. *KRAS^G12D^* PDAC-derived EV-packaged circPNIT facilitates axonogenesis and PNI.** (**A**) Schematic illustration of circPNIT-silencing in PANC-1 and AsPC-1 cells generated via the CRISPR/Cas9 approach. (**B** and **C**) qRT‒PCR analysis of the expression of circPNIT in stromal cells treated with EVs from *KRAS^G12D^* PDAC cells as indicated. (**D**) Quantification of flow cytometric analysis of EVs taken up by stromal cells in the TME treated with PKH67-labeled AsPC-1-EV_WT_ or AsPC-1-EV_circPNIT-KO_ cells. (**E**) Quantification of flow cytometric analysis of EV uptake by DRG cells treated with PKH67-labeled AsPC-1-EV_Vector_, AsPC-1-EV_circPNIT_ or AsPC-1-EV_circPNIT_+αCD109. (**F**-**G**) qRT‒PCR analysis of axonogenesis-related up-regulated genes expression in EV-treated DRG as indicated. (**H**-**I**) qRT‒PCR analysis of DSCAML1 expression in DRG cells treated with EVs as indicated. (**J**) Transcriptional activity of DSCAML1 in DRG cells treated with truncated DSCAML1 promoter luciferase plasmids and EVs derived from AsPC-1 cells. (**K**) ChIRP assays were used to detect circPNIT-associated chromatin fragments in the DSCAML1 promoter in DRG cells. (**L**) Luciferase activity in DRG cells treated with DSCAML1-P3 promoter mutation luciferase plasmids and EVs derived from AsPC-1 cells. (**M**-**N**) ChIP‒qPCR assay of H3K9Ac enrichment on the DSCAML1 promoter in DRG cells treated with EVs derived from AsPC-1 cells as indicated. (**O**) qRT‒PCR analysis of PNI-related gene expression in EV-treated DRG cells as indicated. (**P**-**Q**) Western blotting of crucial proteins in the GFRα1/RET and MAPK/ERK signaling pathways in DRG cells treated with EVs derived from AsPC-1 cells as indicated. Statistical significance was assessed using a 2-tailed Student’s t test in Figures B-D, G, J-L, and N-O. One-way ANOVA followed by Dunnett’s test was used in Figures E-F, H-I, and M. The data are presented as the mean ± SD of three independent experiments. **P*<0.05, ***P*<0.01.


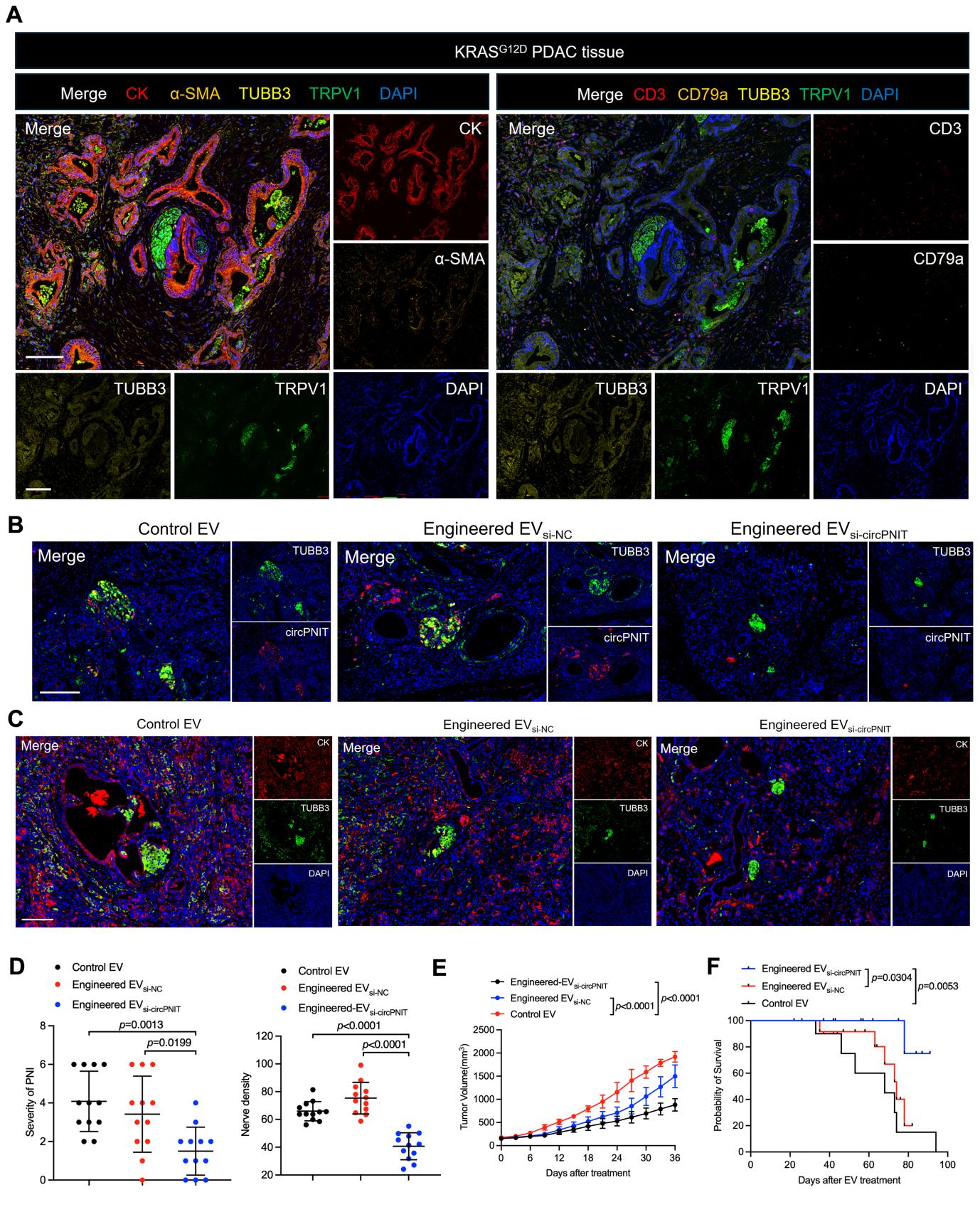


**Figure S9. Targeted inhibition of circPNIT with engineered EVs blocks PNI in *KPC* mouse model.** (**A**) The distribution of TRPV1 in *KRAS^G12D^* PDAC tissue. Scale bars: 50 μm. (**B**) Representative images of circPNIT engagement in KPC tumor tissue after treatment with EV as indicated. Scale bars: 50 μm. (**C-D**) Representative mIHC images (C) and quantification (D) of PNI severity and nerve density. Scale bar: 50 μm. One-way ANOVA followed by Dunnett’s test was used. (**E**) Tumor volumes of KPC model after treated with indicated EVs. (**F**) The survival days of KPC mouse after treated with indicated EVs. Statistical significance was assessed using a One-way ANOVA followed by Dunnett’s test was used in Figures D-E. The data are presented as the mean ± SD of three independent experiments. **P*<0.05, ***P*<0.01.


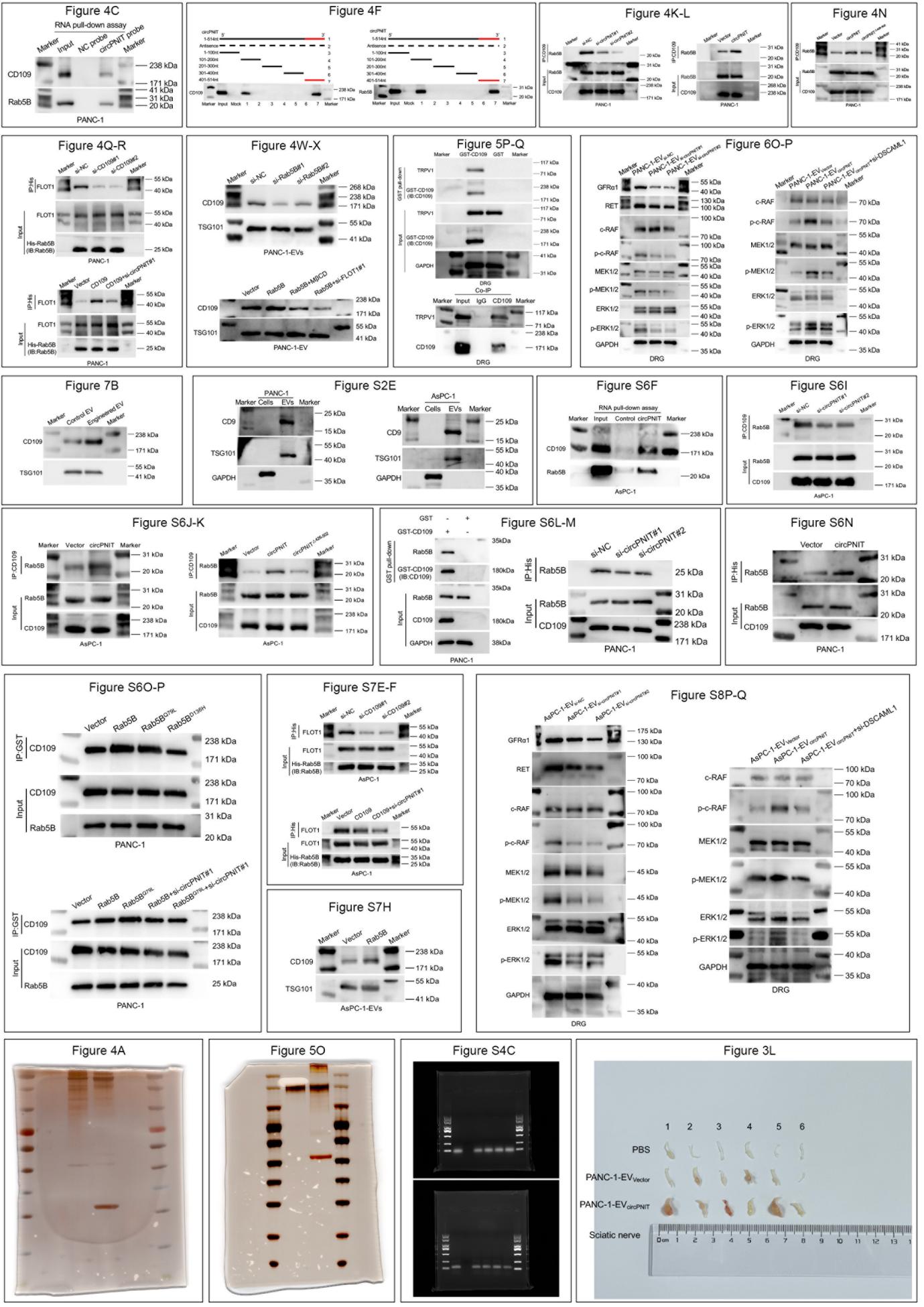


**Figure S10. Full uncut original pictures.**

**Supplemental Tables**

| **Table S1. Correlation between circPNIT expression and clinicopathologic characteristics of *KRAS^G12D^* PDAC patients (*n* = 266) from multi-centers.** | | | | |
| --- | --- | --- | --- | --- |
| **Characteristics** | **N** | **circPNIT expression level** | | |
|  |  | **Low** | **High** | ***P*-value^A^** |
| **Total cases** | 266 | 133 | 133 |  |
| **Gender** |  |  |  | 0.536 |
| Male | 151 | 78 | 73 |  |
| Female | 115 | 55 | 60 |  |
| **Age** |  |  |  | 0.903 |
| ≤60 | 125 | 63 | 62 |  |
| >60 | 141 | 70 | 71 |  |
| **Differentiation** |  |  |  | 0.856 |
| Poor | 61 | 30 | 31 |  |
| Moderate | 172 | 85 | 87 |  |
| Well | 33 | 18 | 15 |  |
| **T stage** |  |  |  | 0.534 |
| T1-2 | 155 | 80 | 75 |  |
| T3-4 | 111 | 53 | 58 |  |
| Perineural invasion |  |  |  | **0.001^**^** |
| Low | 76 | 55 | 21 |  |
| High | 190 | 78 | 112 |  |
| **TNM stage** |  |  |  | 0.550 |
| Stage I | 53 | 30 | 23 |  |
| Stage II | 160 | 78 | 82 |  |
| Stage III | 53 | 25 | 28 |  |
| Abbreviations: N: Number of cases; A: Chi-square test, ^*^*P*<0.05, ^**^*P*<0.01. | | | | |

| **Table S2. Univariate and multivariate analyses of Overall Survival (OS) for circPNIT expression in *KRAS^G12D^* PDAC patients (*n* = 266) from multi-centers.** | | | | | | |
| --- | --- | --- | --- | --- | --- | --- |
| **Variables** | **Univariate analysis** | | | **Multivariate analysis** | | |
|  | **HR** | **95%CI** | ***P*-value^A^** | **HR** | **95%CI** | ***P*-value^A^** |
| Gender (Male vs. Female) | 1.093 | 0.798-1.497 | 0.580 |  |  |  |
| Age (＞60 vs. ≤60) | 1.240 | 0.904-1.702 | 0.183 |  |  |  |
| Differentiation (poor and moderate vs. well) | 1.407 | 0.864-2.289 | 0.170 |  |  |  |
| T stage (T3-4 vs. T1-2) | 1.164 | 0.849-1.597 | 0.346 |  |  |  |
| TNM stage (AJCC) (Stage III and Stage II vs. Stage I) | 1.657 | 1.073-2.559 | **0.023^*^** | 1.623 | 1.048-2.512 | **0.030^*^** |
| Perineural invasion (High vs. Low) | 1.920 | 1.332-2.769 | **<0.001^**^** | 1.479 | 1.004-2.178 | **0.048^*^** |
| circPNIT expression (High vs. Low) | 2.212 | 1.610-3.039 | **<0.001^**^** | 1.987 | 1.421-2.779 | **<0.001^**^** |
| Abbreviations: HR: hazard ratio; 95%CI: 95% confidence interval; T stage: tumor stage; TNM stage: tumor node metastasis stage; A: Cox regression analysis, ^*^ P <0.05, ^**^ P <0.01. | | | | | | |

| **Table S3. Univariate and multivariate analyses of Disease-Free Survival (DFS) for circPNIT expression in *KRAS^G12D^* PDAC patients (*n* = 266) from multi-centers.** | | | | | | |
| --- | --- | --- | --- | --- | --- | --- |
| **Variables** | **Univariate analysis** | | | **Multivariate analysis** | | |
|  | **HR** | **95%CI** | ***P*-value^A^** | **HR** | **95%CI** | ***P*-value^A^** |
| Gender (Male vs. Female) | 1.229 | 0.835-1.429 | 0.521 |  |  |  |
| Age (＞60 vs. ≤60) | 1.263 | 0.965-1.654 | 0.090 |  |  |  |
| Differentiation (poor and moderate vs. well) | 1.144 | 0.771-1.697 | 0.505 |  |  |  |
| T stage (T3-4 vs. T1-2) | 1.281 | 0.980-1.673 | 0.070 |  |  |  |
| TNM stage (AJCC) (Stage III and Stage II vs. Stage I) | 1.333 | 0.945-1.879 | 0.101 |  |  |  |
| Perineural invasion (High vs. Low) | 1.996 | 1.462-2.725 | **<0.001^**^** | 1.657 | 1.202-2.284 | **0.002^**^** |
| circPNIT expression (High vs. Low) | 2.224 | 1.700-2.909 | **<0.001^**^** | 1.977 | 1.499-2.6608 | **<0.001^**^** |
| Abbreviations: HR: hazard ratio; 95%CI: 95% confidence interval; T stage: tumor stage; TNM stage: tumor node metastasis stage; A: Cox regression analysis, ^*^ *P* <0.05, ^**^ *P* <0.01. | | | | | | |

| **Table S4. Primers and probes used in the experiments.** | | |
| --- | --- | --- |
| **Gene** | **Sequence (5’-3’)** | **Application** |
| DYM | F: GAATAGCAGCAGAATCGGCG  R: CTGCTAGTTGGTGCAGGGAA | qRT-PCR |
| circPNIT | F: TCTCCTGATCCTGGTGGTAA  R: GGGAAAGGACTGCTATCTGA | qRT-PCR |
| U1 | F: CAGGGGAGATAACGTGACCA  R: GGGAAAAGCACGGACACAG | qRT-PCR |
| 18s rRNA | F: AGCAGACATTGACCTCACCA  R: CCTCTATGGGCCCGAATCTT | qRT-PCR |
| NTN1 | F: TGACGGGTATCACCTGCAAC  R: AGGAATCGCAGTCTTCAGGC | qRT-PCR |
| IST1 | F: ACTGCCAAAGGGACCATCAG  R: GGGGGAGTTGCTGGTATCTG | qRT-PCR |
| MYCBP2 | F: CGGTCAACGTCACCAAAACC  R: TACCACATGGGGAGAGGGAG | qRT-PCR |
| MACF1 | F: GGGTCGAAGGTCCAAACCAT  R: CTGATAGGGGTGCCAGCTTT | qRT-PCR |
| ITGB1 | F: GTCGTGTGTGTGAGTGCAAC  R: AGACACCACACTCGCAGATG | qRT-PCR |
| USP9X | F: CAATGCAGGCAATGAGCCAG  R: AGGCTGTTGGAATCAAGGCA | qRT-PCR |
| CHL1 | F: ACAGTTAACAGTTCAAATTCCATCA  R: CTGTGGAGTTGGCAAGCCT | qRT-PCR |
| FLRT2 | F: CGCGGTGATATTTGTGCTGG  R: GTGTAGCGCCCCTTTTTGTG | qRT-PCR |
| PLXNA4 | F: TGCAGACGGACATCCATGAG  R: GGTAGCCCGGGACCTCAA | qRT-PCR |
| FLRT3 | F: GGAGCATCTTCCTCATCGGG  R: AGCGACACACAGATGGACAG | qRT-PCR |
| SEMA3C | F: GGATCAGCCGTGTGTGTGTA  R: GCTCCTCCTGGACAAGTTCC | qRT-PCR |
| BMPR2 | F: AGGAGCTGTGAACTTGAGGG  R: TCTGGTACGGATTCCCCTGG | qRT-PCR |
| VEGFA | F: CGAAAGCGCAAGAAATCCCG  R: CTCCAGGGCATTAGACAGCA | qRT-PCR |
| SPP1 | F: ACAAATACCCAGATGCTGTGGC  R: ACTTGGAAGGGTCTGTGGGG | qRT-PCR |
| PRICKLE1 | F: TTATGGCCTGCAGAACCCAG  R: CGGAAGAGGAGGAGGAGGAA | qRT-PCR |
| SEMA6A | F: TCTTACAACACAGTGTATGGGCA  R: GCTGTCAGGTGAGTCAAGCA | qRT-PCR |
| KIF5B | F: GGCCGAGTGCAACATCAAAG  R: CGATCACGACCGTGTCTTCT | qRT-PCR |
| DSCAML1 | F: CTCTTTACACAAAGCCCGCC  R: CACGGAGCTGGAAAAGGTCA | qRT-PCR |
| DSCAML1-P1 | F: GTAGAAACCGGGCGTCCTT  R: GGCTCCGCTGTCTGAGAG | qRT-PCR |
| DSCAML1-P2 | F: GGGTAAGCTGAGTCCGAAAG  R: CTGTCACCCACCGGCTTG | qRT-PCR |
| DSCAML1-P3 | F: GGGTGGGTAGGTGAAGACAA  R: TACTCCCCACCCCAAAGTTC | qRT-PCR |
| DSCAML1-P4 | F: CACACCTTAGAGTCTGCCCA  R: GCCAGGTCTGCTGTAAAAGG | qRT-PCR |
| DSCAML1-P5 | F: CGGTGAGAGATGGGAGCTAA  R: AGGCATAGAACAGAGACGGG | qRT-PCR |
| NGF | F: CCCCCTTCAACAGGACTCAC  R: GAGAATTCGCCCCTGTGGAA | qRT-PCR |
| GDNF | F: GCGCTGAGCAGTGACTCAAA  R: GCCGATTCCGCTCTCTTCTA | qRT-PCR |
| GFRα1 | F: TGTCGGGCAATACACACCTC  R: CTTGGAGGAGCAGCCATTGA | qRT-PCR |
| RET | F: GTGTACGACGAGGACGACTC  R: TCCTTCCGCTTGAACTCCAC | qRT-PCR |
| Artemin | F: TGGAACTTGGACTTGGAGGC  R: GAGAGACCAAGTGGAGCCTG | qRT-PCR |
| NTRK1 | F: CCCTCTGTACCCCCGATCTT  R: TCTCGATGTAGCTTGCTGCC | qRT-PCR |
| NGFR | F: CACCGACAACCTCATCCCTG  R: CTATGTAGGCCACAAGGCCC | qRT-PCR |
| CXCL12 | F: CTACAGATGCCCATGCCGAT  R: CAGCCGGGCTACAATCTGAA | qRT-PCR |
| CXCR4 | F: TCCATTCCTTTGCCTCTTTTGC  R: ACGGAAACAGGGTTCCTTCA | qRT-PCR |
| CX3CL1 | F: GGCACCTTCGAGAAGCAGAT  R: CACAGACTCGTCCATTCCCC | qRT-PCR |
| CX3CR1 | F: TCGTGGTCTTTGGGACTGTG  R: TGTTGGTGAGGGCAAACACT | qRT-PCR |
| SEMA3A | F: GCAGCTGATTTTATGGGGCG  R: TGCTGCTCTGTCCTGATTGG | qRT-PCR |
| PLXND1 | F: GTATCGGCCGCAGATCATGG  R: CTTGTGCTGCAGTTGTGTCC | qRT-PCR |
| GAPDH | F: AATGGGCAGCCGTTAGGAAA  R: GCGCCCAATACGACCAAATC | qRT-PCR |
| ACTB | F: AGAGCCTCGCCTTTGCC  R: GGGGTACTTCAGGGTGAGGA | qRT-PCR |
| circPNIT-sgRNA1 | GAGAATGACGTGAACCCAGG | CRISPR-Cas9 |
| circPNIT-sgRNA2 | AGTGAGCTGAGATCGCACCA | CRISPR-Cas9 |
| si-circPNIT#1 | sense: GGAUCAUCAGAUAGCAGUCCUdTdT  antisense: AGGACUGCUAUCUGAUGAUCCdTdT | siRNA |
| si-circPNIT#2 | sense: AUCAUCAGAUAGCAGUCCUUUdTdT  antisense: AAAGGACUGCUAUCUGAUGAUdTdT | siRNA |
| si-Rab5B#1 | sense: GCUGCAAUCGUGGUUUACGACAUUA  antisense:UAAUGUCGUAAACCACGAUUGCAGC | siRNA |
| si-Rab5B#2 | sense: GCAAUCGUGGUUUACGACAUUACUA  antisense:UAGUAAUGUCGUAAACCACGAUUGC | siRNA |
| si-CD109#1 | sense: CGCUUAUCAUUUGAGACCAAGAGAA  antisense:UUCUCUUGGUCUCAAAUGAUAAGCG | siRNA |
| si-CD109#2 | sense: AAGAGAAUAUCUGUCUUCAUUCAAA  antisense:UUUGAAUGAAGACAGAUAUUCUCUU | siRNA |
| si-FLOT1#1 | sense: CAGUCACUGGCAUUGCCCAGGUAAA  antisense:UUUACCUGGGCAAUGCCAGUGACUG | siRNA |
| si-FLOT1#2 | sense: CGGGAAGCUAAAGCCAAGCAGGAAA  antisense: UUUCCUGCUUGGCUUUAGCUUCCCG | siRNA |
| si-DSCAML1#1 | sense:CCUCUUUAUGGCAUGUGGCUGGUAA  antisense:UUACCAGCCACAUGCCAUAAAGAGG | siRNA |
| si-DSCAML1#2 | sense:CCCUUCAUGUGACCCUGACACCAAA  antisense:UUUGGUGUCAGGGUCACAUGAAGGG | siRNA |
| AGO2-sgRNA1 | CCGCTGCCTCTTCAGAGAAG | CRISPR-Cas9 |
| AGO2-sgRNA2 | TGAGACGGTCTCTGTTGACC | CRISPR-Cas9 |
| si-TRPV1#1 | sense: ACGAAGUUUGUGACGAGCAUGUACA  antisense:UGUACAUGCUCGUCACAAACUUCGU | siRNA |
| si-TRPV1#2 | sense: CGAGCAUGUACAAUGAGAUUCUGAU  antisense:AUCAGAAUCUCAUUGUACAUGCUCG | siRNA |
| circPNIT | GGAAAGGACUGCUAUCUGAUGAUCCUCUGGGC | Pulldown |
| circPNIT | GGAAAGGACUGCUAUCUGAUGAUCCUCUGGGC 5’-Cy3 labeled and 3’-Cy3 labeled | ISH |
| U1 | CGCAGGGGTCAGCACATCCG  5’-Cy3 labeled and 3’-Cy3 labeled | FISH |
| 18s rRNA | CTTCCTTGGATGTGGTAGCCGTTTC  5’-Cy3 labeled and 3’-Cy3 labeled | FISH |

| **Table S5. Antibodies used in the experiments.** | | |
| --- | --- | --- |
| **Product** | **Source** | **No. of Catalogue** |
| ***Western blot:*** |  |  |
| anti-CD109 | Invitrogen | 14-1099-80 |
| anti-Rab5B | Proteintech | 27403-1-AP |
| anti-6× His | Proteintech | 66005-1-Ig |
| anti-FLOT1 | Proteintech | 15571-1-AP |
| anti-CD9 | Proteintech | 20597-1-AP |
| anti-TSG101 | Abcam | ab133586 |
| anti-GFRα1 | Abcam | ab8026 |
| anti-RET | Abcam | ab134100 |
| anti-c-Raf | Cell Signaling Technology | 9422T |
| anti-p-c-Raf | Cell Signaling Technology | 9427T |
| anti-MEK1/2 | Cell Signaling Technology | 8727T |
| anti-p-MEK1/2 | Cell Signaling Technology | 9154T |
| anti-ERK1/2 | Cell Signaling Technology | 9102S |
| anti-p-ERK1/2 | Cell Signaling Technology | 4370T |
| anti-GAPDH | Proteintech | 60004-1-Ig |
| anti-TRPV1 | Abcam | ab305299 |
| ***IHC:*** |  |  |
| anti-TUBB3 | Proteintech | 66375-1-Ig |
| anti-DSCAML1 | Abcam | ab150761 |
| ***IF/mIHC:*** |  |  |
| anti-pan-CK | Abcam | ab7753 |
| anti-TUBB3 | Proteintech | 66375-1-Ig |
| anti-Rab5B | Proteintech | 27403-1-AP |
| anti-CD109 | Invitrogen | 14-1099-80 |
| anti-CD63 | Proteintech | 25682-1-AP |
| anti-EEA1 | Proteintech | 68065-1-Ig |
| anti-Rab7 | Cell Signaling Technology | 9367T |
| anti-LAMP2 | Cell Signaling Technology | 34141S |
| anti-FLOT1 | Proteintech | 15571-1-AP |
| ***IP:*** |  |  |
| anti-CD109 | Invitrogen | 14-1099-80 |
| anti-GST | Proteintech | HRP-66001 |
| anti-TRPV1 | Abcam | ab305299 |
| anti-6× His | Proteintech | 66005-1-Ig |
| anti-H3K9Ac | Active Motif | 91104 |
| **Secondary antibody:** |  |  |
| ***Western blot:*** |  |  |
| anti-rabbit IgG-HRP | Proteintech | SA00001-2 |
| anti-mouse IgG-HRP | Proteintech | SA00001-1 |
| ***IHC:*** |  |  |
| anti-rabbit IgG-HRP | Proteintech | SA00001-2 |
| anti-mouse IgG-HRP | Proteintech | SA00001-1 |
| ***IF:*** |  |  |
| [Alexa Fluor 555](https://www.baidu.com/link?url=nF9d2Xaur7vyZuSh6bwYgXJHxCoqgi5ljmVkB6q--I4j4E8mmfQwWu1WHii3mT9LmMQ5XQE23xsmXGJKAUgtmctb7vuX9L1odcCwYmhMNRgtKDKfaFBBlPGw7dU_i5LpQii0iVI7_AAuccqxBIw54_&wd=&eqid=cad6a7aa0006a5040000000661054afd) | Abcam | ab150074 |
| [Alexa Fluor 4](https://www.baidu.com/link?url=nF9d2Xaur7vyZuSh6bwYgXJHxCoqgi5ljmVkB6q--I4j4E8mmfQwWu1WHii3mT9LmMQ5XQE23xsmXGJKAUgtmctb7vuX9L1odcCwYmhMNRgtKDKfaFBBlPGw7dU_i5LpQii0iVI7_AAuccqxBIw54_&wd=&eqid=cad6a7aa0006a5040000000661054afd)88 | Abcam | ab150113 |
| AST-487 | MCE | HY-15002 |

**Supplemental Methods**

***Measurement of nerve density***

DRG cells were cocultured with 20 µg of EVs for 48 h, fixed with 4% paraformaldehyde, and stained with TUBB3 and DAPI. Nerve density was measured by counting neurons in four random fields at 20× magnification, and the density of nerve axons was calculated by the total number of axonal branches divided by the total number of nucleus.

***Isolation of DRG cells***

Cell culture plates were precoated with poly-L-lysine at 37 °C and 5% CO_2_ for 1 h and washed three times with PBS for DRG to adhere. Four-week-old nude mice were killed by intraperitoneal injection of pentobarbital anesthesia, and the spine was exposed while the mice were in the prone position. After the longitudinal incision of the spine and removal of the spinal cord, the DRG were extracted. Add 1 ml 0.2% IV collagenase and 0.25% trypsin into DRG, and digested at 37 °C for 20 minutes. The digestion solution was blown through until the tissue was amorphous. Then, terminated by 2 ml of FBS. After filtration through 100 µm and 40 µm cell filters, the cells were cultured in DMEM overnight at 37 °C and 5% CO_2_. The next day, 5 μM cytarabine was added to removing proliferating cells.

***Transwell assay***

2×10^5^ DRG cells were preseeded in a 24-well tissue culture plate. The transfected PDAC cells were added into the chamber that was pre-coated with or without Matrigel (BD biosciences, NY, USA) and each chamber was added 100μl serum-free culture medium and placed in a 24-well tissue culture plate. After 18 h of incubation, the invaded cells were fixed and stained. All images were captured with the microscope (Olympus, Tokyo, Japan) and the number of migrating cells from upper chamber were measured by ImageJ software (ImageJ, RRID:SCR_003070) in 5 random fields.

***PET-CT***

PET-CT was used to evaluate orthotopic tumors in nude mice. Four-week-old nude mice were fasted for 8 h and anesthetized with inhaled pentobarbital. 5 Ci/g of ^18^FDG was dissolved in 50ul of 0.9% saline and injected via the tail vein of nude mice. PET-CT scan was performed 30 min after the ^18^FDG injection. ^18^FDG uptake within the tumor was calculated.

***Isolation and purification of EVs***

EVs were isolated from cell culture media or fresh tissues using differential centrifugation. The cell culture media were collected after 48 h of transfection. Fresh tissues were digested with collagenase I in FBS-free DMEM at 37 °C for 20 min. The cell culture medium and tissue digests were centrifuged at 2,000 × g for 20 min to remove the cells and debris. The supernatant was collected and centrifuged at 10,000 × g for 40 min to remove the microvesicles. After filtration, the supernatant was further centrifuged at 120,000 × g for 70 min, and the pellets were washed in PBS by centrifugation at 120,000 × g for another 70 min. The pelleted EVs were resuspended in 20 µl of PBS and stored at 4 °C for further analysis. The EVs from the serum samples were extracted using an exosome isolation kit (Thermo Fisher Scientific, USA) following the manufacturer’s instructions. The protein content of the EVs was quantified using a BCA Protein Assay Kit (Pierce, Rockford, IL, USA). EV-packaged RNA was extracted by TRIzol.

***Characterization of EVs***

For transmission electron microscopy (TEM), the isolated EVs were deposited on the grid for 60 min and fixed with 2.5% glutaraldehyde for 10 min. The grids were washed with PBS five times, followed by incubation with uranyl acetate for 5 min. Finally, the grids were washed with PBS, dried, and imaged using a Hitachi transmission electron microscope (HITACHI, Tokyo, Japan).

For nanoparticle tracking analysis (NTA), the pellets of EVs were diluted in PBS to obtain concentrations up to 2 ×10^9^ particles/ml. The concentration and size of the EVs were assessed using a NanoSight LM10 (Malvern, Framingham, MA) by recording for 30 s with a 488 nm laser at 25 °C. Analysis was performed using NTA v3.1 software (Malvern, Framingham, MA).

For immunoelectron microscopy analysis, EVs were blocked with 10% FBS for 30 minutes. After that, the EVs were incubated with an anti-CD109 antibody (Invitrogen, USA; 14-1099-80) overnight at 4 °C. The next day, anti-mouse IgG bound to 12 nm gold particles was added to the mixture, which was subsequently incubated for 1 h at room temperature and washed with PBS. Next, 1% neutral glutaraldehyde was added to the EVs, which were incubated for 5 min and washed with ultrapure water. The EVs were embedded in a mixture of 4% uranyl acetate and 2% methylcellulose at a ratio of 1:9 and observed using JEOL transmission electron microscopy (JEOL, Tokyo, Japan) at 80 kV.

For nanoflow cytometric analysis, EVs were incubated with an anti-CD109 antibody (Invitrogen, USA; 14-1099-80) for 1 h at room temperature, washed with PBS, and incubated with an anti-mouse fluorescent secondary antibody (Alexa Fluor 488; Abcam, ab150113) for 1 h. Subsequently, the EVs were gated with mixtures of 100 nm, 200 nm (Horizon), and 500 nm yellow-green beads (Polysciences). Unstained EVs, isotype-stained EVs, and antibodies alone were used as negative controls. Nanoflow cytometry analysis was conducted with a CytoFLEXS (Beckman Coulter, USA). FlowJo v10 (BD Biosciences, NY, USA) was used for data analysis.

***Loading of si-circPNIT into Engineered EVs***

To package si-circPNIT into CD109^+^EV, 50 μg CD109^+^EV and 50 μg si-circPNIT were dissolved in 90 μl of cold electroporation buffer containing 21% Opti-MEM™ Reduced Serum Medium, 1.15mM pH 7.2 potassium phosphate and 25mM potassium chloride. Then, the mixture was transferred to a 4mM cuvette and electroporated 20 times at 0.35s pulses and 0.7kV voltage. The mixture was then incubated at 37°C for 30 minutes to restore the full membrane structure. To remove free si-circPNIT, the mixture was washed twice with cold PBS, and the precipitate was obtained by ultraculcentrifugation at 120,000g for 70 min at 4 °C and resuspended in PBS. Si-circPNIT was loaded into CD109^+^EVs and the mixture was divided into two groups. One group was incubated with RNase at 37°C for 30 minutes to degrade the siRNA on the surface of CD109^+^EVs (Rafter digestion), and the EVs without RNase was used as a control (Rbefore digestion). To eliminate electroporation-induced siRNA accumulation and RNase tolerance, we used circPNIT without CD109^+^EV equivalent as a negative control for electroporation (Rcontrol). The quantification of siRNA was measured by serial dilutions of FITC-siRNA of known concentration using a microplate reader (Biotek) with an excitation wavelength of 488 nm and an emission wavelength of 518 nm, which were used to plot a standard curve. The CD109^+^EV-loaded si-circPNIT and the supernatant were collected and the 488nm and 518nm wavelengths were measured separately. The concentration of siRNAs in the samples was estimated from the standard curve. siRNA loading efficiency was calculated as (%) = (Rafter digestion - Rcontrol)/( Rbefore digestion)×100%.

***In vitro and in vivo assessment of EV uptake***

EVs were labeled with PKH67 according to the instructions of the PKH67 green fluorescent labeling kit (Sigma‒Aldrich, USA, MINI67), and the excess dye was neutralized using 5% BSA. Then, the PKH67-labeled EVs were assessed by ultracentrifugation. In vitro, EVs were incubated with DRG cells for 6 h. After washing three times with PBS, the plates were fixed with 4% paraformaldehyde for 15 min and stained with DAPI for 5 min. In vivo, 50 μg of EVs were injected into the pancreatic tissue of nude mice every 5 days. At the end of the experiment, the tumor tissues were collected for immunofluorescence staining. The CD109 neutralizing antibody (αCD109) was synthesized by HUABIO (Zhejiang, China). The images were taken under a Zeiss confocal microscope system (Carl Zeiss MicroImaging, Germany).

***Multiplex immunohistochemistry (mIHC)***

mIHC staining was performed using a PANO 4-plex IHC Kit (Panovue, China) according to the manufacturer’s suggested protocols. Briefly, after deparaffinization and rehydration, the paraffin slides were placed in an EDTA buffer (pH 9.0) for heat-induced antigen retrieval using a microwave. After incubating in goat serum (Golden Bridge Biological Technology, China) at room temperature for 10 min, the slides were incubated with primary antibodies for 1 h, followed by incubation with horseradish peroxidase-conjugated secondary antibodies and tyramine signal amplification. The slides were heated after each tyramide signal amplification operation. DAPI was used to stain the nuclei. Images were obtained using a Zeiss LSM900 confocal microscope (Carl Zeiss MicroImaging, Germany).

***Immunohistochemistry (IHC)***

Pancreatic cancer tumor tissues and xenograft model tumor tissues were fixed with 4% paraformaldehyde and analyzed via IHC. Briefly, the sections were dewaxed with dimethylbenzene and hydrated in gradient alcohol solutions. Subsequently, the antigens were retrieved using EDTA, and the sections were blocked in goat serum and then incubated with primary antibodies at 4 °C overnight. After secondary antibody incubation, the location of the antigens was determined using a DAB substrate (Thermo Fisher Scientific, Cat# 34002). Hematoxylin staining was used to determine the nuclear morphology. All images were captured with a microscope (Olympus, Tokyo, Japan).

***In situ hybridization (ISH)***

An Enhanced Sensitivity ISH Detection Kit II (Boster Biological Technology, MK1032) was used to detect the expression of circPNIT in PDAC tissues embedded in paraffin. After deparaffinization and hydration, the tissue sections were digested with pepsin to fully expose the RNA fragments and hybridized with a digoxin (DIG)-labeled circPNIT probe overnight at 37 °C. Subsequently, the sections were labeled with an anti-digoxin antibody for 2 h at 37 °C. Finally, BCIP/NBT (5-bromo-4-chloro-3-indolyl phosphate/nitroblue tetrazolium) staining and nuclear fast red counterstaining were performed. All images were captured with a microscope (Olympus, Tokyo, Japan).

***Flow cytometry***

After enrichment with 2×10^5^ stromal cells, the cells were fixed and ruptured by using FIX & PERM™ media (Thermo Fisher Scientific, USA, GAS004) for 20 minutes. Subsequently, the cells were incubated with primary and fluorescent secondary antibodies for 20 min at room temperature and washed with PBS, after which the target proteins were detected via a Beckman CytoFLEX (Beckman Coulter, USA).

***RNA pull-down assays***

RNA pull-down assays were performed to investigate circPNIT binding proteins in *KRAS^G12D^* PDAC cells. First, the biotinylated circPNIT probe and NC probe were incubated with PDAC cells lysate at 4℃ overnight. Subsequently, the elution protein was analyzed by silver staining or western blot, and the differential bands were detected by mass spectrometry.

***Serial deletion analysis and site-directed mutagenesis***

circPNIT with various deletions were amplified by using a series of paired 3’ nested primers with common 5’ primers or 5’ nested PCR primers with common 3’ primers and cloned into pcDNA3.0 for in vitro transcription. The RNAs probe were constructed for further validation in RNA pull-down assays. The mutant circPNIT RNAs (Δ451-502,Δ426-477,Δ426-502,) described in the manuscript were synthesized following the instruction of QuikChange Site-directed Mutagenesis Kit (Stratagene, CA, USA).

***RNA immunoprecipitation (RIP) assays***

RIP assays were also conducted to confirm the interaction of circPNIT with Rab5B and CD109 by using an EZ-Magna RIP Kit (Merck, Darmstadt, Germany; Cat# 17-701). Briefly, 2×10^7^ PDAC cells were lysed in RIP lysis buffer at -80 °C for 3 hours. Then, the cell lysate was centrifuged at 120,000 × g for 10 min, after which the supernatant was collected and incubated with magnetic beads conjugated with anti-CD109 (Invitrogen, 14-1099-80, USA) or anti-His (His-Rab5B; Proteintech, 66005-1-Ig, China) antibodies at 4 °C overnight. The retrieved RNAs were eluted and further subjected to qRT‒PCR analysis.

***Co-IP assays***

Co-IP assays were conducted according to the manufacturer’s instructions for the Co-Immunoprecipitation Kit (Pierce, Rockford, IL, USA; Cat#26149). Briefly, 1 × 10^7^ PDAC cells were lysed in a lysis buffer. The cell lysate was added to an amine-reactive resin coupled with antibodies, and the mixture was immunoprecipitated at 4 °C overnight. The eluted proteins were further analyzed using mass spectrometry and western blot analysis. Normal rabbit/mouse IgG was used as the negative control.

***Luciferase reporter assays***

The luciferase reporter assays were performed to detect the regulation of circPNIT on DSCAML1 promoters. Briefly, we inserted different fragments of the DSCAML1 promoters upstream of the luciferase gene promoter region to construct the pGL3 reporter plasmid and transfected it into DRG cells. After 48h incubation, the luciferase activity was measured by a luciferase reporter assay kit (Promega, Madison, WI, USA, Cat#E1910).

***ChIRP assays***

Following the protocol of the Magna ChIRP RNA Interactome Kit (BersinBioTM, China, Catalog Bes5104-3), ChIRP assays and qRT‒PCR analysis were performed to detect interactions between EV-packaged circPNIT and its target gene promoters. DRG cells (2 × 10^7^) were induced with 10 μg/mL EVs for 48 h, fixed with 40 ml of PBS containing 1% paraformaldehyde for 15 min, and lysed in cell lysis buffer. The cell lysate was then ultrasonicated at 4 °C for 1 hour to obtain fragments of 100 to 200 bp. Subsequently, the biotinylated circPNIT probes were added and incubated at 4 °C overnight. The next day, agarose beads were added to extract the DNA for qRT-PCR analysis.

***Chromatin immunoprecipitation (ChIP) assays***

We used an EZ-Magna ChIP A/G kit (Millipore, Billerica, MA, USA; Cat# 17-371) to detect the interaction of H3K9Ac with the DSCAML1-promoter DNA fragments. A total of 1 × 10^7^ DRG cells were collected and cross-linked in PBS containing 1% formaldehyde for 10 min at room temperature. Subsequently, the cells were lysed with cell lysate or nuclear lysate to obtain chromatin. Then, the chromatin was sheared into 500 to 800bp fragments by ultrasonication and hybridized with an anti-H3K9Ac antibody (Active Motif, 91104). The chromatin fragments bound with the anti-H3K9Ac antibody were immunoprecipitated with protein A/G-coated magnetic beads at 4 °C overnight. The DNA was purified and analyzed by quantitative real-time PCR (qRT–PCR). Normal mouse immunoglobulin G (IgG) was used as a negative control.

***GST pull-down***

To obtain total protein samples, 500 μl of lysis buffer was added to 1 × 10^7^ DRG cells, which were lysed at room temperature for 30 min and centrifuged at 12000 × g for 30 min. Then, the lysate was incubated with purified GST-tagged CD109 overnight at 4 °C. The next day, anti-GST antibody-conjugated magnetic beads were added to pull down and elute the target protein for subsequent analysis.

***Proximity ligation assays (PLA)***

The interaction between CD109 and TRPV1 was detected by PLA. Briefly, 3×10^4^ DRG cells were fixed on confocal dishes and incubated overnight in a serum-free medium. After that, the DRG cells were fixed with 4% paraformaldehyde for 15 min and blocked with 5% BSA for 1 hour. Subsequently, PLA was performed with a DUOLINK PLA Kit (Sigma‒Aldrich, Germany) according to the manufacturer’s instructions. The interaction between CD109 and TRPV1 was observed via laser scanning confocal microscopy (Carl Zeiss MicroImaging, Germany).

***The CRISPR/Cas9 approach***

A pair of sgRNAs targeting the AGO2 or DYM coding sequence were designed by the CRISPR Guide RNA Design Tool (<https://www.benchling.com/crispr>). Subsequently, sgRNAs targeting AGO2 or DYM were cloned and inserted into plasmids and transfected into PANC-1 and AsPC-1 cells. Knockdown efficiency was determined by western blot and qRT-PCR.

***Plasmids and siRNA transfection***

The plasmids and siRNAs were designed and constructed by Igebio (Guangzhou, China). Then the plasmids or siRNAs were transfected into PDAC cells with Lipofectamine 3000 Kit (Invitrogen, USA, Cat#L3000015) according to the instructions of manufacture.

***Lentivirus infection***

A total of 1×10^5^ cells were added to a 6-well plate the day before infection. DMEM supplemented with 10 µg/ml polyprene was added to the lentivirus stock solution to prepare a lentivirus working solution. One milliliter of lentivirus working solution was added to each well of the plate. Eight hours later, the lentivirus working solution was discarded, and the cells were cultured in DMEM for 72 hours. Puromycin (10 µg/mL; Solarbio, Beijing, China; Cat# P8230) was applied to the cells to screen for successfully transfected cells.

***Western blot assays***

The protein sample was added to the gel, and the voltage was adjusted from 65 V to 150 V when the protein sample reached the separation gel. After the target protein was separated, the gel was removed, the proteins were combined with polyvinylidene fluoride (PVDF), and the proteins were transferred to the membrane at a constant pressure of 25 V for 15 min (Bio-Rad Trans-blot Turbo, USA, 170415). The PVDF membrane was blocked with 10% BSA for 1 hour, and the membrane was incubated with primary antibodies at 4 °C overnight. After the membranes were washed with TBST three times, the secondary antibodies were incubated at room temperature for 1 hour. The protein bands were detected using an enhanced chemiluminescence (ECL) kit (Pierce, NE, USA; Cat# 32106).

***qRT-PCR analysis***

The total RNA of PDAC cells was extracted following the instruction of the Universal RNA Purification Kit (EZBioscience, China, EZB-RN4) and reverse transcribed by Hiscript III Reverse Transcriptase kit (Vazyme, Nanjing, China, Cat#R312-01). The corresponding RNA expression was evaluated by qRT-PCR with ChamQTM Universal SYBR qPCR Master Mix kit (Vazyme, Nanjing, China, Cat#Q711-02).

***RNase R treatment and actinomycin D assays***

Total RNA was extracted following the protocol of the Universal RNA Purification Kit (EZBioscience, China, EZB-RN4). Total RNA (2 μg) was mixed with 3 U/μl RNase R (Geneseed Biotech, China, Cat# R0301) at 37 °C for 30 min. The equivalent RNA without RNase R was used as a control. The expression of circPNIT and DYM mRNA was evaluated by qRT‒PCR.

For actinomycin D assays, 1×10^5^ PANC-1 or AsPC-1 cells were cultured in a 6-well plate overnight. PDAC cells were treated with 5 μg/ml actinomycin D (APExBIO, Houston, TX, USA; Cat# A4448) at the same time points, after which the PDAC cells were collected at specific time points (0, 6, 12, 18 and 24 h) and total RNA was extracted. The expression of circPNIT was evaluated by qRT‒PCR.

***Subcellular fractionation assays***

According to the instructions of the PARISTM Kit (Thermo Scientific, USA, Cat# AM1921), 1×10^6^ PDAC cells were incubated with cell membrane lysis buffer for 10 minutes. After centrifugation at 500 × g and 4 °C for 5 min, the supernatant was transferred to new microtubes to obtain the cytoplasmic lysate. Then, 300 µl of nuclear lysis buffer was added to the precipitate. Then, 2× lysis/binding solution and anhydrous ethanol were added to the cytoplasmic lysis solution and the nuclear lysis solution, the mixture was passed through a transfer column, and the separation column was washed. The separation column was transferred to a new collection tube. Then, 40 µl elution buffer was added, after which 10,000 g were centrifuged at 4℃ for 30 s and the RNA sample was collected.
